# Uniqueness of Protected Areas for Conservation Strategies in the European Union

**DOI:** 10.1038/s41598-018-24390-3

**Published:** 2018-04-24

**Authors:** Samuel Hoffmann, Carl Beierkuhnlein, Richard Field, Antonello Provenzale, Alessandro Chiarucci

**Affiliations:** 1 Department of Biogeography, BayCEER, University of Bayreuth, Universitaetsstr. 30, D-95440 Bayreuth, Germany; 2 School of Geography, University of Nottingham, University Park, Nottingham, NG7 2RD UK; 3 Institute of Geosciences and Earth Resources, National Research Council of Italy, Via Moruzzi 1, 56124 Pisa, Italy; 4 Department of Biological, Geological, and Environmental Sciences, Alma Mater Studiorum – University of Bologna, Via Irnerio 42, 40126 Bologna, Italy

## Abstract

Protected areas (PAs) constitute major tools in nature conservation. In the European Union (EU), the Birds and Habitats Directives are the most important policies for conservation strategy, legally preserving Europe’s characteristic, rare, endemic and threatened biota. We used occurrence data for species listed in the directives’ Annexes to assess the uniqueness of major PAs in the EU (National Parks, Biosphere Reserves); this is important for preserving the EU’s focal species. We developed a novel, multifunctional approach to calculate different metrics of conservation value that represent different components of species diversity within the PAs, involving inventory diversity, deviation from the species–area relationship, species rarity and differentiation diversity. Applying it, we found that individual PAs frequently vary considerably in their scores on different components, which are often disconnected from PA size. PAs around the EU periphery, often containing few species, are key to conserving species that are rare in the EU. Because our analysis focuses on EU priority species and includes different components of diversity, it allows more appropriate estimation of conservation value inside PAs in context of the EU than recent, high-profile, global-level research. We offer tools to evaluate, and information to regulate, the representativeness, persistence and efficiency of PAs.

## Introduction

Beyond climate change, biodiversity decline is considered the major threat to human well-being in the 21^st^ century^1^. In 2010, the Earth’s nations agreed again to try to halt biodiversity loss by 2020 (Aichi Biodiversity Targets^2^), but global prospects of improvement are still slight^3^. The effectiveness of conservation action has still to increase^4^.

Protected areas (PAs) represent a fundamental tool in nature conservation policies, their main purpose, often achieved, being to conserve local to regional biodiversity, particularly the characteristic or threatened species, habitats and ecosystems^5–7^. Often, PAs are the only remaining safe sites for species’ populations^8^, whose existence relies on PA performance. However, the Convention on Biological Diversity^2^ predominantly relates to the PA surface area, stating: “By 2020, at least 17 per cent of terrestrial and inland water areas and 10 per cent of coastal and marine areas […] are conserved through effectively and equitably managed, ecologically representative and well-connected systems of protected areas” (Target 11 of Aichi Biodiversity Targets). There is thus a risk of naively focusing on the amount of area, but neglecting biodiversity protection^9,10^.

The contribution of PAs to preventing habitat loss and maintaining biodiversity is debated^5,11,12^. Studies reveal poor management effectiveness^13^, growing human pressures^14^ and insufficient governmental support^15–17^. Changes to PAs themselves often affect their conservation role, including species invasions, pollution, acidification, nitrogen deposition and climatic change^14,18–21^.

The efficiency of PAs has been studied at several scales. We refer to ‘scale’ as the geographical extent of the study region: ‘global scale’ as cross-continental extent, ‘regional scale’ as cross-national to continental extent, and ‘local scale’ to national or smaller extent. Many investigations of PA performance focus on local scales, but regional and global biodiversity cannot be maintained by a few isolated PAs^17,22,23^. Therefore, regional and global gap analyses have been applied to suggest strategies to complete protection networks^11,24–26^, but gaps are unlikely to be filled if only local criteria and policies matter^27^. Moreover, local, regional and global conservation priorities often differ greatly, and the performance of PA networks strongly depends on the geographical context they are applied to^11,28–30^. It is also questioned whether the global distribution and geographical density of PAs satisfy the conservation needs in the regional context^31^. Conservation effort also differs between local and global extents; continental-scale approaches are therefore considered a reasonable compromise to evaluate the real capacity for biodiversity protection of existing PA systems^17,32^, particularly in Europe, where human population density is high and the legacies of land use, settlements and infrastructures allow little freedom for PA extension. Cultural landscapes and anthropogenic ecosystems (e.g. hay meadows) characterize European nature and PAs^33,34^.

Since conservation aims and monitoring are primarily set for individual areas, comparing PAs’ performance on larger scales is challenging. However, in the European Union (EU), the Birds and Habitats Directives are legally binding conservation policies. They enforce member states to protect and report spatial records of many characteristic, endangered, vulnerable, rare and endemic species (but see Lisón *et al*.^35^) that are listed in the Annexes of the directives. We refer to these priority species as ‘reported species’. The directives also form the legal basis for the Natura 2000 PA system, which has global importance^12,22,36,37^. The reported species are focal species for conservation at the political level of the EU.

Here, we use reported species data from the Birds and Habitats Directives^38^ to identify the individual contribution of renowned European PAs to preserve species diversity. We thus concentrate on conservation prioritization by the EU for the EU. We measure different indices to account for various components of the conservation value of PAs, including uniqueness. We do not evaluate the uniqueness of PAs against unprotected areas; we treat the PAs as self-operating and isolated sites that are, assuming the worst case, the last remaining safe sites for biodiversity in future. We focus on national parks (NP) and UNESCO Man and Biosphere Reserves (MAB), because they have particularly significant benefits for biodiversity conservation in the EU due to their large areas, and integrative, intensive and effective management of biological objectives^5,7,17,34,39,40^. In total, 285 NPs, 147 MABs and 1303 species in ten taxa are considered herein, with the incidence data of species based on 10 km × 10 km grid cells. To assign species’ occurrences to PAs, we developed a probabilistic approach based on the overlap of grid cells and PA polygon area.

In conservation research, uniqueness is often measured only as rarity^41^. Our evaluation is manifold, rather than relating to a single concept. We measure inventory diversity (both directly and accounting for the species–area relationship [SAR]), species rarity and differentiation diversity. To measure the PAs’ conservation value in these ways, we calculate seven indices (Table 1), producing a multifaceted estimation of the conservation value of major PAs for protecting focal EU species, which contrasts with a recent global approach^23^. We also perform sensitivity analyses to assess the reporting deficits of individual EU member states and how these affect measures of conservation value. The sensitivity analyses include a null model approach that compares the observed values within individual EU states to values from a random model, and to the observed values of all other EU states. Moreover, we conduct cross-validation to estimate sensitivity to reporting bias. Our methodological approaches are generally suitable for conservation assessment involving other components of biodiversity, different PAs and geographic extents. The results can guide future conservation effort to enhance the persistence and efficiency of biodiversity preservation inside single PAs and PA networks.

**Table 1:** Metrics of conservation value applied in this study. For details about indices’ definitions and the distributions of data for them, see Methods and Results. All the indices apply to units of space (protected areas [PA]) except conservation weight, which applies to individual species (reported species [RS]).

Name	Abbreviation	Description
Reported Species Richness	*Richness_RS*	Estimated number of reported species, calculated as the sum of the species’ probabilities of occurrence (based on overlap between the PA and grid cells occupied by each species).
Area-controlled Surplus of Reported Species	*Richness_SAR_%Surplus*	Residual from modelling the species–area relationship (SAR) for the protected areas, expressed as percentage of the modelled value. Thus, if the expected *Richness_RS* of a PA based on its area is 50 and the actual *Richness_RS* is (a) 70 or (b) 35, then its *Richness_SAR_%Surplus* is (a) (70–50)/50 = 40% or (b) (35–50)/50 = −30%.
Conservation Weight	*w*	Inverse of the total number of grid cells occupied by the species in the land area of the EU. It estimates species rarity in the EU.
Rarity-weighted Richness	*Richness_Rarity_weighted*	Sum of the products resulting from the multiplication of the species’ probabilities of occurrence by the species’ conservation weights. It integrates reported species richness and rarity.
Average Rarity	*Rarity_Mean*	Average conservation weight of the reported species present (*Richness_Rarity_weighted/Richness_RS*).
Total Dissimilarity	*Dissimilarity_Total*	Overall dissimilarity of species composition between protected areas, calculated using the method of Baselga (2013). It can be additively partitioned into *Dissimilarity_Balanced* and *Dissimilarity_Gradient* as described below. The dissimilarity score of a single PA is the mean of all pairwise dissimilarity values of the PA compared with all others.
Balanced Dissimilarity	*Dissimilarity_Balanced*	Dissimilarity in terms of balanced gains and losses (i.e. turnover) of species abundances between sites, calculated using the method of Baselga (2013).
Gradient Dissimilarity	*Dissimilarity_Gradient*	Dissimilarity in terms of monotonic gradients of species abundances (gains or losses) between sites, calculated using the method of Baselga (2013).

## Results

### Metrics of Conservation Value

**Figure 1:**
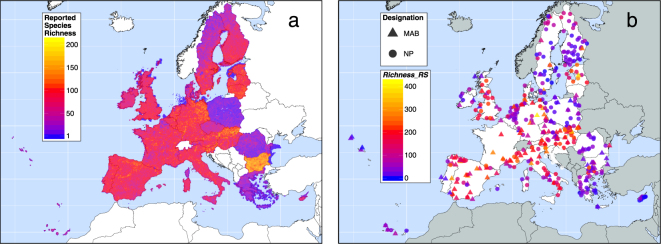
(**a**) Reported richness of the 1654 Annex species of the Birds and Habitats Directives per 10 km × 10 km grid cell in the European Union. The 41 marine species are excluded. (**b**) Reported species richness within 285 national parks (NP) and 147 Man and Biosphere Reserves (MAB) in the European Union. The values estimate the number of Annex species of the Birds and Habitats Directives within these protected areas. For details see Methods section. The maps were created using open-source software R, Version 3.3.3 (https://www.R-project.org/)^60^.

Reported species richness in PAs broadly reflects the richness pattern in grid cells (Fig. 1), except that most PAs in Bulgaria have relatively low values. Variation in reported species richness across EU member states is apparent. The richness of reported species per grid cell (range 0–189) appears low in Poland, the Czech Republic, Romania and Greece, and remarkably high in Bulgaria (but see the ‘Sensitivity Analyses’ section). We find unexpectedly low reported bird species richness in Poland, the Czech Republic, Romania and Greece (see also Supplementary Fig. S1).

**Figure 2:**
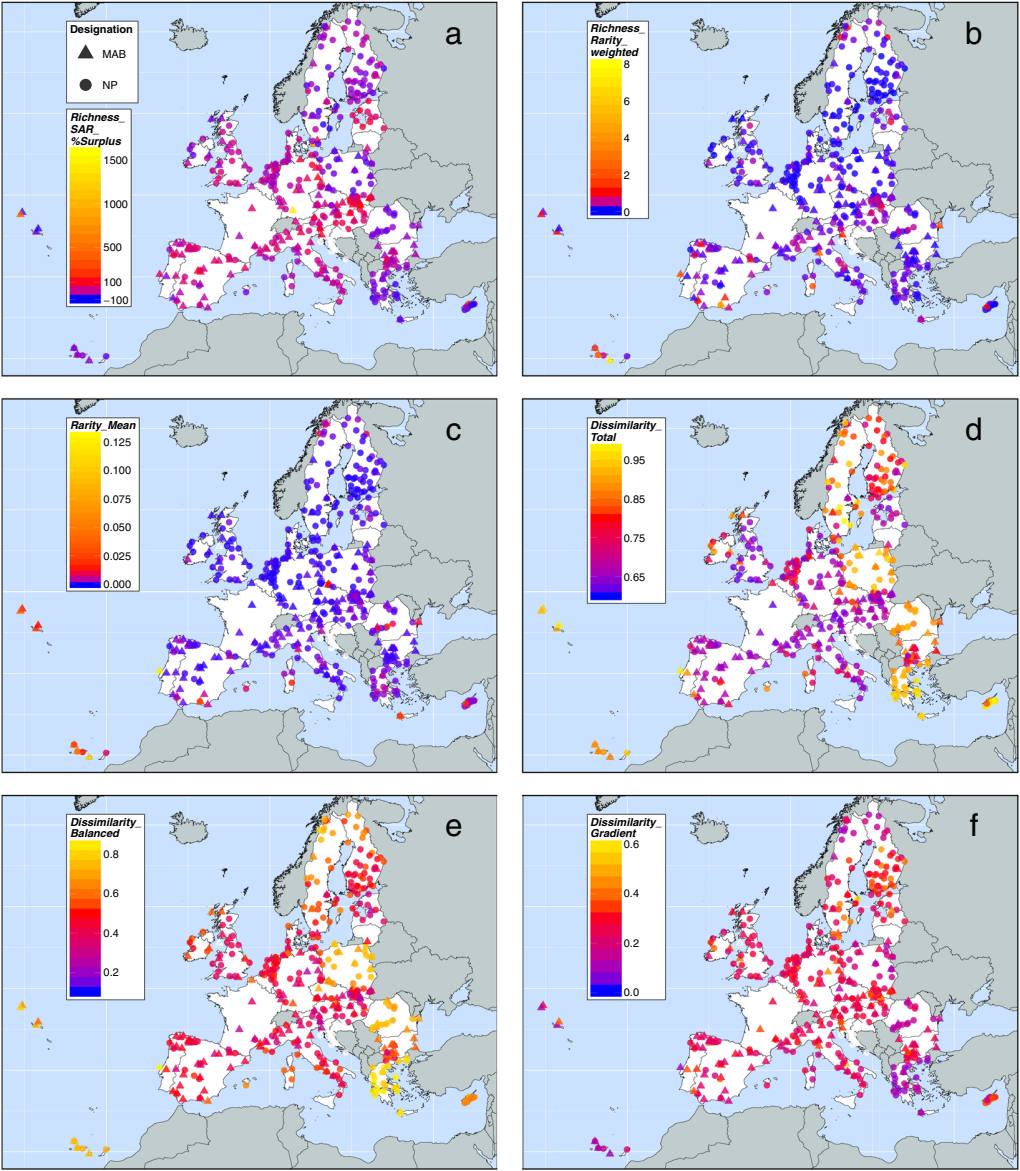
Metrics of conservation value for national parks (NP) and UNESCO Man and Biosphere Reserves (MAB) in the European Union. (**a**) Area-controlled surplus of reported species (*Richness_SAR_%Surplus*) accounts for the effect of area on reported species richness. It reveals the percentage deviation between observed *Richness_RS* and predicted *Richness_RS*, as modelled by the species–area relationship considering observed reported species richness and protected area. (**b**) Rarity-weighted richness (*Richness_Rarity_weighted*) integrates reported species richness and rarity. It is a measure of the protected area’s reported species richness, but weighted by the conservation weights of reported species. (**c**) Average rarity (*Rarity_Mean*) is calculated by *Richness_Rarity_weighted* over *Richness_RS*. It represents the average rarity of reported species within the protected area. (**d**) Total dissimilarity (*Dissimilarity_Total*) indicates beta diversity between protected areas regarding their species composition. (**e**) Balanced dissimilarity (*Dissimilarity_Balanced*) and (**f**) gradient dissimilarity (*Dissimilarity_Gradient*) are the additive components of total dissimilarity (Baselga, 2013). For details about indices’ definition see Methods section. The maps were created using open-source software R, Version 3.3.3 (https://www.R-project.org/)^60^.

**Figure 3:**
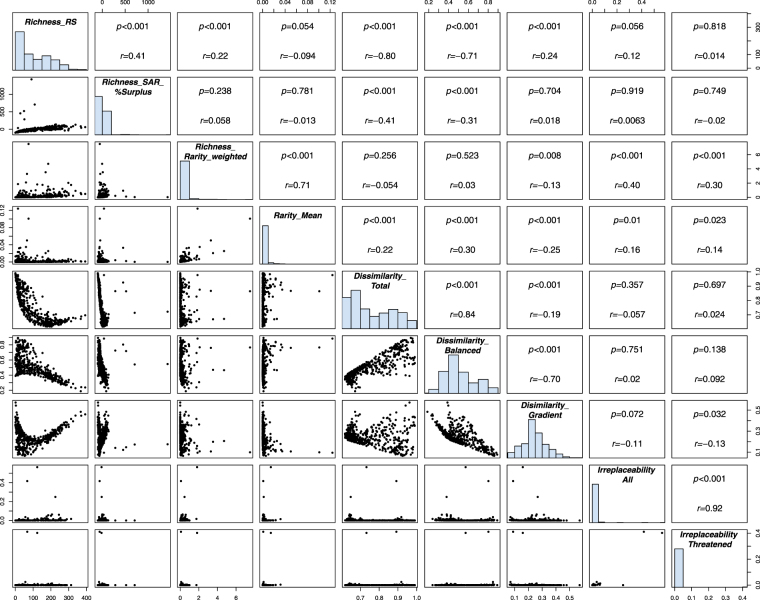
Correlations among metrics of conservation value of protected areas: reported species richness (*Richness_RS*), rarity-weighted richness (*Richness_Rarity_weighted*), average rarity (*Rarity_Mean*), area-controlled surplus of reported species (*Richness_SAR_%Surplus*), total dissimilarity (*Dissimilarity_Total*), balanced dissimilarity (*Dissimilarity_Balanced*), gradient dissimilarity (*Dissimilarity_Gradient*), irreplaceability for all species (*Irreplaceability All*) and irreplaceability for threatened species (*Irreplaceability Threatened*). Irreplaceability values were calculated by Le Saout *et al*.^23^. The *r* indicates the Pearson correlation coefficient, whereas *p*-values reflect the significance of the correlation considering spatial autocorrelation. Panels at the diagonal show frequency distributions of the variables.

The other metrics of conservation value (area-controlled surplus of reported species, rarity-weighted richness, average rarity, total dissimilarity balanced dissimilarity and gradient dissimilarity; Fig. 2) only partially correlate with reported species richness (Fig. 3). For example, Eastern European countries tend to have low values for most of these metrics, but high values of compositional dissimilarity. Macaronesian islands have high values for uniqueness-related metrics. High uniqueness scores are often found for clusters of PAs, especially around the periphery of the EU.

Many PAs with lower reported richness than expected from their areas (negative area-controlled surplus of reported species) are in states known for low reported richness (Fig. 2a). In other EU regions, also, some PAs have such reported species deficits, for example on Macaronesian islands, in the Mediterranean Basin, in the United Kingdom, Sweden and Finland. Scattered across the EU are some PAs with strongly positive surpluses of reported species for their sizes (e.g. in Estonia, Latvia, Germany, Slovakia, Hungary, Austria, Slovenia, Bulgaria and Spain).

**Figure 4:**
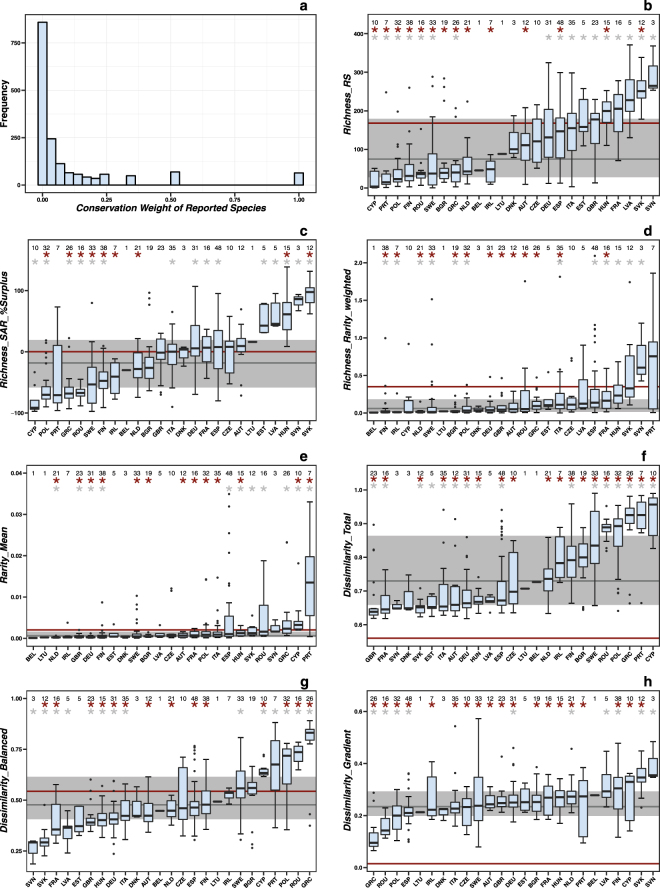
Sensitivity analysis to assess potential reporting bias of EU member states. (**a**) Frequency distribution of species’ conservation weight *w*, where frequency is the number of species. For Annex species of the Birds and Habitats Directives, *w* was calculated as the inverse of the sum of grid cells in which the species is present, within the land area of the EU. The conservation weights of reported species were maintained for the randomization procedure included in this sensitivity analysis for (**b**) *Richness_RS*, (**c**) *Richness_rarity_weighted*, (**d**) *Rarity_Mean*, (**e**) *Richness_SAR_%Surplus*, (**f**) *Dissimilarity_Total*, (**g**) *Dissimilarity_Balanced*, and (**h**) *Dissimilarity_Gradient*. In b) to h) the thick black line within blue boxes represents the median. The limits of the blue box show the lower and upper quartiles (interquartile range). The whiskers extend to the lowest to the highest values within 1.5 times the interquartile range. The black dots indicate outliers (beyond the whiskers). The horizontal grey line depicts the median of the EU-wide distribution that was observed based on the original data. The corresponding grey strip shows the interquartile range of this observed distribution. The grey stars show significant differences (*p* < 0.05) between the observed values within an EU member state (ISO3 code) and the rest of the EU. The horizontal red line shows the median of the EU-wide distribution that was simulated by randomization. The narrow red strip represents the interquartile range of this simulated distribution. The red stars indicate a significant difference (*p* < 0.05) between the observed and randomized values per EU member state. The black number above each boxplot gives the number of data points (PAs) per EU member state. The boxplots are ordered by their median, increasing from left to right. Transboundary protected areas were excluded. For details see Methods.

The values of rarity-weighted richness (<0.1–7.5) are heterogeneously distributed across EU member states (Fig. 2b). Single PAs with high rarity-weighted richness are found on Macaronesian islands, in the Mediterranean Basin, around the Black Sea, in parts of Central Europe, the Baltic region and in Northern Scandinavia. In most of the rest of the EU, PAs have low rarity-weighted richness. PAs with the highest average rarity tend to occur where rarity-weighted richness is also high (Fig. 2c). The range of average rarity values (<0.01–0.12) suggests that average reported species rarity is low within the PA network; reassuringly, most reported species are relatively common in the EU’s PAs (Fig. 4a).

Total compositional dissimilarity is generally high (0.62–0.99), but is particularly high in many PAs containing few reported species (Fig. 2d). Total dissimilarity is partly decoupled from reported species richness; for example, in southern Sweden, PAs containing high reported species richness are also very dissimilar in species composition. Balanced dissimilarity, indicating species turnover between PAs, shows an analogous pattern (Fig. 2e). Nestedness-resultant dissimilarity among PAs (gradient dissimilarity; Fig. 2f) contributes less to total compositional dissimilarity than the turnover component. Furthermore, total dissimilarity of PAs with low reported species richness in Poland, Czech Republic, Romania and Greece is mainly composed of turnover-resultant dissimilarity; these PAs tend to host relatively few reported species that are unique to these areas. The standard deviation of pairwise dissimilarity values of a single PA is typically low for PAs that have high mean dissimilarity scores (total dissimilarity) and vice-versa (Supplementary Fig. S2). For a complete list of the PAs and their conservation-related metrics, see Supplementary Information.

The relationships between the metrics of conservation value (Fig. 3) reinforce the geographical patterns described above. The index ‘area-controlled surplus of reported species’ is strongly associated with reported species richness (*r* = 0.41, p < 0.001). Rarity-weighted richness is more strongly related to average rarity (*r* = 0.71) than to reported species richness (*r* = 0.22), though both correlations are significant (p < 0.001; Fig. 3). Reported species richness is negatively related to total dissimilarity; this relationship is strong and non-linear (Fig. 3). Total dissimilarity is much more weakly, and positively, associated with average rarity (*r* = 0.22, p < 0.001), which suggests a small influence of species rarity on compositional dissimilarity. High balanced dissimilarity corresponds to high total dissimilarity (*r* = 0.84, *p* < 0.001), which indicates that dissimilarity is dominated by species turnover.

### Sensitivity analyses

The sensitivity analyses estimate potential reporting bias of EU member states and how this influences the PAs’ conservation values. The results generally show (Fig. 4b–h) that the distribution of observed values in the EU (associated with the grey line and wide grey strip) is very different from the random distribution (red line and narrow red strip). Thus, the Annex species are non-randomly distributed within the entire set of PAs. The reported species richness values in Poland, Romania and Greece are significantly (i.e. *p* < 0.05) lower than (i) randomly expected (red stars, Fig. 4b), and (ii) in the remaining EU states (grey stars). This supports the visually perceived paucity of reported (bird) richness in Poland, Romania and Greece (Fig. 1a, Supplementary Fig. S1), but not in the Czech Republic. For Bulgarian PAs, not only did we detect not significantly higher reported richness than in the rest of the EU, but the richness values are actually lower than expected under random species distribution. We also identified EU states that include PAs of significantly low (Cyprus, Portugal, Finland and Sweden) and high reported richness (Hungary and Slovakia), but these do not show extreme reported richness per grid cell (Fig. 1a). Other nations have *Richness_RS* values that are neither significantly distinct from randomization, nor from the rest of the EU, which does not support the existence of a bias. Moreover, the observed *Richness_RS* values are generally much lower than under the randomized null model.

Very similar results were found for the area-controlled surplus of reported species (Fig. 4c), because PA size was maintained in the null model. Furthermore, these sensitivity patterns vary little when the species’ conservation weights are involved (i.e. *Richness_Rarity_weighted*, Fig. 4d), since in the null model the weights were maintained as well. The conservation weights barely changed the *Richness_RS* pattern, because most conservation weights are low (Fig. 4a). These findings are in line with sensitivity results for average rarity (Fig. 4e), which is strongly correlated to rarity-weighted richness (Fig. 3).

The total dissimilarity of PAs is, in general, significantly higher than randomly expected (Fig. 4f). *Dissimilarity_Total* is primarily driven by species turnover (Figs 3 and 4g) rather than nestedness (Figs 3 and 4h). Slovakia and Hungary show significantly lower total dissimilarity than the remaining EU states, while Finland, Sweden, Romania, Poland, Greece, Portugal and Cyprus indicate significantly higher values. This conforms to the sensitivity results of *Richness_RS*. The PAs of Finland, Sweden, Romania, Poland, Greece, Portugal and Cyprus include significantly fewer as well as different species than are found in the majority of other PAs. Slovakia contains PAs that are not only rich in reported species, but also significantly more similar to other PAs than are the remaining PAs in the EU. Therefore, many reported species of Slovakian PAs also occur in many other PAs that include fewer species. Hungary presents similar relationships, but turnover-resultant dissimilarity dominates here. Note that in some EU states the number of PAs was not sufficient to adequately test for significant differences between value distributions.

**Figure 5:**
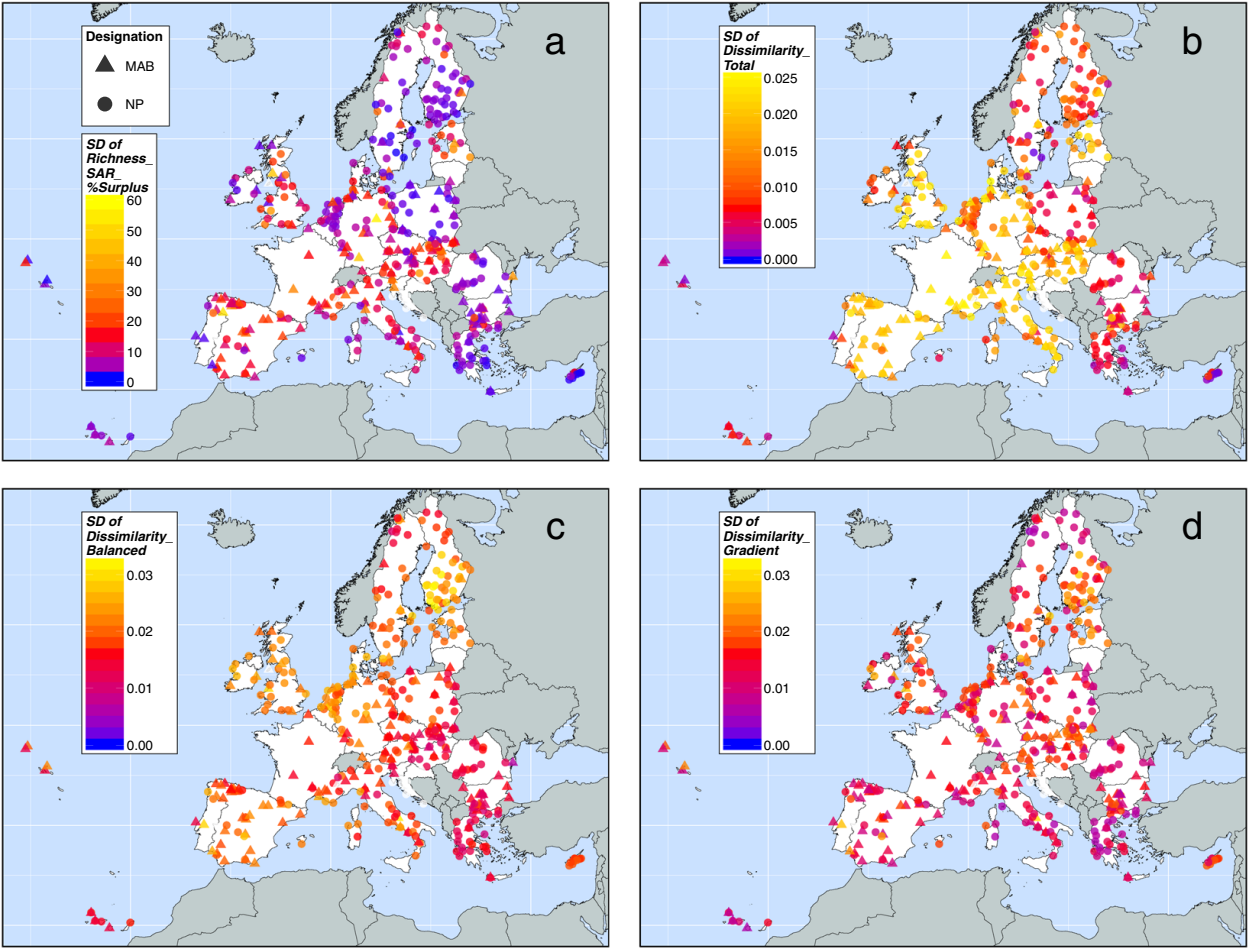
Cross-validation of four metrics of conservation value against potential reporting failure of EU member states. Five different nations were randomly excluded in each of 1,000 runs, to simulate the absence of biased countries and its effect on uniqueness indices. The standard deviation (SD) of resulting uniqueness values is a measure of uncertainty under reporting failure. In other words, it represents the robustness of conservation values of protected areas against potential reporting deficits of EU member states. Since we assume a lack of reported species only, we maintained the species’ conservation weights in this sensitivity analysis (for details see methods section). Thus, only (**a**) area-controlled surplus of reported species, (**b**) total dissimilarity, (**c**) turnover and (**d**) nestedness are affected by this simulation procedure. Transboundary protected areas were excluded. The maps were created using open-source software R, Version 3.3.3 (https://www.R-project.org/)^60^.

The cross-validation of area-controlled surplus of reported species and dissimilarity indices indicates how robust the PAs’ conservation values are against potential reporting deficits of EU member states (Fig. 5). Under simulated absence of species records and PAs of various nations, the dissimilarity metrics show a small amount of variation in relation to their absolute values. The *Richness_SAR_%Surplus* index shows larger relative variation. The conservation values of many PAs in Central, Western, and Southern Europe are less stable although we did not detect extraordinary conservation values in these nations. The extreme conservation values of Eastern and Western European states that we identified before, are more stable. This suggests that the conservation values of these nations are distorting the absolute conservation values of the remaining countries even if the relative deviations are small; continental patterns of uniqueness values are sustained.

### Irreplaceability and the Metrics of Conservation Value

**Figure 6:**
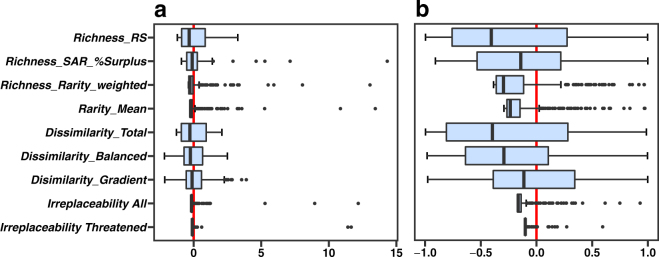
The standardized value range of metrics of conservation value for national parks (NP) and UNESCO Man and Biosphere Reserves (MAB) in the European Union. The value range of metrics is standardized to zero mean (red line) and unit variance (horizontal axis). Boxplots as in Fig. 4. The black dots indicate outliers. (**a**) The total ranges of the values. (**b**) Zoomed in, to display only from −1 and 1 standard deviations from the mean.

The irreplaceability scores obtained from Le Saout *et al*.^23^ have weak positive correlations with the rarity-related metrics rarity-weighted richness and average rarity only (Fig. 3), and these correlations are driven by only a very small number of PAs. Moreover, the amount of variation in the scores from Le Saout *et al*.^23^ is very limited for these European PAs (Figs 3 and 6).

### Separating National Parks, Biosphere Reserves and Taxa

We detected significant differences between the distributions of *Richness_SAR_%Surplus* values of the combined set of PAs versus the separated sets (Supplementary Fig. S3), but geographical patterns remained similar. The same is true for *Dissimilarity_Total* (Supplementary Fig. S3). Hence, *Richness_SAR_%Surplus* and dissimilarity indices are sensitive to the selection of PAs. The metrics of conservation value also depend on the species involved. When single taxa were considered separately (Supplementary Figs S1 and S4 to S12), the uniqueness values of single PAs frequently differed from results for all taxa.

## Discussion

A macroscopic perspective best guides a comprehensive conservation strategy^11,12,20,23^. Surprisingly, however, little research effort has aimed to demonstrate how biodiversity is distributed among European PAs^22^, though most nature conservation funding by the EU has not been directed towards regions with urgent conservation needs^32^. With our study, we provide the first analytical approach to estimate and compare different components of species diversity across the set of European NPs and MABs. The results provide a quantitative, holistic assessment of conservation value of PAs and can form a basis for decision-making, conservation prioritization and targeting future field research. Funding strategies require transparent instruments to set conservation priorities for the spatial distribution of conservation effort^16,33,37,42^. Our approach enables PAs to be rated and compared, with respect to biodiversity components of conservation concern, as represented by reported species, and can be easily applied to different datasets and PA types. The results are based on data with intrinsic limitations, but represent a first and unique attempt to assess the conservation capacity of major European PAs for priority species of the EU.

The study outcomes can support EU-wide conservation planning by demonstrating strengths and weaknesses of the set of European NP and MAB sites. We found consistently high uniqueness on Macaronesian islands, the Mediterranean Basin, Northern Scandinavia and parts of Central and Eastern Europe. The uniqueness of PAs in these regions is driven by the rarity of reported species, but most of these PAs contain relatively few species. This demonstrates a potential management conflict regarding the overall conservation value of these PAs, because it is not trivial to decide whether it is more valuable to preserve many common or few rare species. Low richness and high rarity of Annex species around the periphery of the EU is probably not only related to distance decay ─ the increase of compositional dissimilarity of PAs with distance between PAs ─ but also to the occurrence of regionally endemic species in these isolated, species-poor regions. Another reason for high uniqueness in some parts of the periphery of the EU (especially eastern and north-eastern EU) is species that mostly occur beyond the EU, whose ranges extend into the EU. Many PAs in those peripheral areas are of relatively little importance for the global conservation of such species, but these PAs do contribute a lot to the conservation of these species within the EU; our study brings the EU-wide conservation effort into focus and identifies responsibilities of PAs and corresponding nations for the conservation of legally protected, priority species inside the EU. Thus, our study does not directly address the conservation needs beyond the EU, but several Annex species are endemic to the EU, implying specific responsibility of the EU to preserve such species at the global scale.

We found that the size of PA is not a strong predictor of reported richness; many PAs contain considerably more or fewer species than expected from their size. The isolation of the Macaronesian islands means relatively low species richness for their sizes, which probably explains the low SAR-related richness values of PAs on those islands. We also found clusters of PAs with distinctly different species composition from the rest, emphasizing the value of the regional perspective. Compositional dissimilarity is a crucial dimension of conservation performance of PA networks^43^ that is widely neglected^44^. It is a distinct and fundamental component of biodiversity that informs about site complementarity, and is therefore highly relevant to multi-site conservation, such as PA networks. Relatively high uniqueness scores are generally more dependent on species composition rather than species richness, as it was indicated by the relationships between conservation indices, including the higher contribution of turnover than nestedness dissimilarity. Note that absolute dissimilarity values are sensitive to the selection of PAs, whereas the relative, continental patterns are not.

Mapping reported species richness per grid cell suggests variation in data quality among countries. We thus suppose under-reporting of species in Poland, Romania and Greece, particularly for birds. The conservation values of PAs in these nations may therefore be underestimated and must be interpreted with care. By applying beta diversity partitioning (i.e. turnover and nestedness), we found, nevertheless, that the reported species of these PAs are very different from the remaining nations. These uniqueness indices and average rarity indicate that rare Annex species occur inside these PAs. The low uncertainties of conservation values in these countries support the idea that these PAs have distinct species composition, despite the reporting deficit. While they bias the absolute conservation values of the remaining PAs, the biogeographical patterns of uniqueness are robust against the reporting deficiency.

In the sensitivity analyses, some other PAs had conservation values very different from expectations based on species richness, with no other indication of reporting bias; we suppose that this is for ecological reasons. The geographically marginal location in the EU, and isolation, may reduce the number of Annex species present in Portugal and Cyprus. The latitudinal richness gradient accounts for low reported richness in Finland and Sweden, and low human impact might enhance species richness found in Hungary and Slovakia. Many PAs in these areas have relatively high uniqueness values suggesting that many Annex species are only found in few nations. Consequently, such nations are especially responsible for the protection of Annex species in the EU and, in case of endemics, at the global scale.

Our evaluation of data deficiencies – especially the lack of (bird) species occurrences in Poland, Romania and Greece – agrees strongly with the data quality evaluation by the EEA^45^. The EEA highlights several nations and sub-nations with serious reported data deficits, but does neither provide details nor reasoning. For Spain, the Canary Islands, Poland, the Azores, Madeira and Romania, over 5% of mandatory information under Article 12 of the Birds Directive is missing and over 25% is noted as “absent” or “unknown”. Mandatory data include species distribution data. With respect to the Habitats Directive (Article 17), only Portugal submitted data in which over 25% of information was marked as “unknown” or “absent”. Data quality information for Greece is missing, probably due to the nation’s delayed submission of data. These facts may explain the exceptional conservation values of PAs in Poland, Romania, Greece and Portugal even though a reporting bias in Portugal was not visually striking. The EEA also states data deficits in Spain; our sensitivity analyses do not support a lack of reported species in Spain, but indicate common conservation values of Spanish PAs. In this case, we suppose that the data deficits do not relate to species distributions. Finally, the data quality evaluation of the EEA supports the interpretation of our data quality evaluation, suggesting that Poland, Romania, Greece and Portugal failed to report the spatial distribution of all Annex species present, but this under-reporting is not enough to substantially affect the EU-wide uniqueness patterns.

Using different methods and data, our findings are partially in line with, but greatly add to, a global study of irreplaceability of PAs^23^. Our study uses a wider range of metrics and more taxa, is more comprehensive within the EU and provides new tools. The uniqueness indices correlate little with the irreplaceability scores calculated by Le Saout *et al*.^23^, which, being globally calibrated, show minimal variation for all but three European PAs, and thus have minimal discriminatory power for the EU. This again emphasizes the value of the EU-wide perspective. Recalculating global-scale conservation indices for regional-scale conservation systems can deliver more meaningful results for the regional context. In consideration of both species richness and rarity, our indices agree in the rating of PAs regarding their potential to protect species considered by the EU directives and the IUCN Red List, respectively.

Biodiversity-based indicators of conservation value strongly depend on the set of species analyzed. It is claimed that species listed in Annexes of the Birds and Habitats Directives were not strategically selected, i.e. their conservation status in the EU was not considered^46^. A few species of the directives are neither threatened in, nor native to Europe, and their European Annex status is not always consistent with the European Red List status. But the Annex species are considered ‘umbrella species’ for many different taxa^37,47^: a lot of other species profit from their protection. We therefore expect that our uniqueness values reflect a much higher proportion of biodiversity than Annex species only. In addition, we neither use the Annex status, nor the Red List status to estimate the conservation value of species in the EU. The conservation weight we applied is solely based on occurrence data, which is a simple and reproducible measure of conservation status with high spatial resolution and a metric scale.

The conservation values we calculated for individual PAs depend not only on the species involved, but also on the study extent. Our analysis quantifies conservation value inside PAs; it does not assess the conservation value of unprotected areas. Protecting all facets of biodiversity at the global extent is the ultimate goal in nature conservation, but depending on future policy and land-use change, the conservation value of protected areas, in themselves, may be crucial. Accordingly, we focus on the PAs as self-operating, isolated units in the European landscape, an approach similar to an important global study of irreplaceability focusing only on PAs and did not include data from their surroundings^23^. Applying our methods to this global data set is possible and offers further research potential, but, as we show, such global-level research can almost completely miss regional-scale patterns. An important question not addressed by our study is: how much does the conservation effort of the EU contribute to global conservation needs?

Staff deficits and financial undersupply are major challenges for European PAs^16^; such local restrictions can cause considerable bias and noise in data directly derived from park and reserve authorities, when conducting large-scale comparisons. This is a key reason why our EU-wide comparison of PAs used the standard set of species that EU member states must report. Additionally, PAs’ corresponding grid cells are likely to be more extensively sampled than unprotected cells, because of monitoring and research activities by PA authorities and other parties^16^. The Birds and Habitats Directives are legally binding regulations, significant and conclusive for nature conservation in the EU. The statutory duty and high importance for conservation across the EU make this dataset unique and expedient for conservation biogeography^37^.

National parks and MAB reserves are cornerstones of the European PA network. Although there are many such PAs, their distribution does not conform to patterns of high biodiversity or rarity. National policies, the history and philosophy of planning, and conservation management have had strong influences on the spatial distribution of PAs^9,11,15^, especially in Europe, where national biases become evident^33,36^. The current situation clearly shows differences in the distribution of NPs and MABs between countries, with large countries containing relatively few (e.g. France) and relatively small countries with many (e.g. The Netherlands). Thus, our research also demonstrates that the development of effective conservation planning at the European scale requires assessment and standardization of PA classification across the European countries, as intended by the IUCN management categories and the Natura 2000 framework, establishing Special Protection Areas (SPAs) under the Birds Directive and Special Areas of Conservation (SACs) under the Habitats Directive.

However, the SPAs and SACs are often small and lack effective management^16,42,45,48,49^. Furthermore, frequent PA designations such as habitat management areas, protected landscapes and areas for the use of natural resources are primarily not established and regulated to protect species. The European NPs and MABs are, in contrast, far from ‘paper parks’, because they protect biodiversity by individual, independent, intensive and integrative management^39,40^ while promoting ecosystem goods and services. Thus, NPs and MABs may be more efficient in implementing new conservation insights in future^17^. Further, funds from recreational visits can be spent on conservation^50^. These are reasons why we selected NPs and MABs to study.

Conservation value goes beyond considering only species diversity; phylogenetic, trait, habitat and ecosystem diversity should be included. However, these are more difficult to address. Evaluating reasons for, and threats to, the measured uniqueness would need to include factors such as isolation, connectivity, anthropogenic pressure and climate change. These aspects remain for future investigations. However, our concept of uniqueness can be easily adopted for data of similar structure (e.g. ecosystem functions and services) and can serve as a common tool to judge the conservation value of PAs.

Biodiversity knows no political borders. Regional-scale nature conservation needs international coordination and implementation of integrative, yet adaptive, conservation policies – PA management plans and species protection programs. The approach we propose merges such policies to evaluate the representativeness of PAs with respect to species conservation inside a PA network. It can be easily adapted for other biodiversity aspects, from genes to ecosystem level, depending on data availability. We thus emphasize the importance of high-quality data for large-scale conservation assessments. Our study may serve as one basis for future conservation action. We encourage national authorities to cooperate and support funding beyond national boundaries to improve the adequacy of nature conservation in view of a rapidly changing world.

## Methods

### Protected Area Network and Reported Species

The PAs we included in the analyses were those within the EU that are designated as either ‘National Parks’ (NPs) or UNESCO ‘Man and Biosphere Reserves’ (MABs). In some cases MABs entirely or partly cover NPs. We therefore conducted analyses for the whole set of PAs, and separately for the NP and MAB networks. We obtained spatial data on PAs from the World Database on Protected Areas^51^ and the UNESCO MAB Biosphere Reserve Directory^40^. Protected areas for which species reporting was at least partly missing (e.g. for Croatia and transboundary PAs at the EU border) were excluded from analyses. In total 432 terrestrial and semi-terrestrial PAs were considered, 285 of which are NPs and 147 are MABs. We excluded purely marine PAs, and marine species. For 7 national parks (NPs) and 120 UNESCO Man and Biosphere Reserves (MABs) boundary data were not available. In this case we used circular buffers of reported PA surface area around given PA centroids (see also Le Saout *et al*.^23^). The PA polygons comprise all parts that officially belong to the PA, such as buffer and core zones. In total, 55 NPs overlap with 53 MABs. Ten of these 53 MABs entirely contain eight NPs. No MAB is entirely enclosed by any NP. Five MABs are identified as transboundary. The quantity of NPs and MABs is remarkably low in France, Lithuania and Belgium (Fig. 1).

We used species occurrence data published by the European Environment Agency in fulfilment of EU legislation^38^. Known locations of Annex species of the Birds Directive (2009/147/EC; Annex 1 to 5) and the Habitats Directive (92/43/EEC; Annex II, IV and V) had to be reported by EU member states for 2008–2012 (under Article 12 of the Birds Directive) and 2007–2012 (under Article 17 of the Habitats Directive). We refer to these as ‘reported species’. The Annexes involve characteristic, rare, endemic, vulnerable and endangered species at the level of the EU ─ not necessarily global ─ that were selected via expert knowledge of a European Committee. Both directives require EU member states to achieve a favourable conservation status of Annex species within the EU. Detailed information about the reported species and their conservation status is provided by the European Topic Centre on Biological Diversity (EIONET)^52^. According to the species lists for the reporting under Article 12 of the Birds Directive^38^, EU member states reported on 576 wild bird species, which are the majority of bird species naturally occurring in the EU. Out of these species, 193 are particularly threatened within the EU, which means prone to extinction, vulnerable to habitat changes, and rare in terms of small population and range size. For these species EU member states must provide ‘Special Protection Areas’ (SPA), which are one type of Natura 2000 site. The other species considered by the Birds Directive are protected through hunting, capture and trade restriction, or are subject to specific research, monitoring and management regimes. Referring to the species lists for the reporting under Article 17 of the Habitats Directive^38^, 1319 species of other taxa must be reported, including fish, amphibians, arthropods, mammals, molluscs, reptiles, vascular and non-vascular plants. They are rare, endemic, vulnerable or threatened in the EU. For these species the EU member states are obliged to manage ‘Special Areas of Conservation’ (SAC), which cover the core habitat of those species. The sites are also part of the Natura 2000 network. Moreover, a strict protection regime must be applied across the range of those species on EU territory. The exploitation of species is also legally restricted for some species listed in the Habitats Directive.

Species reporting covered all EU member states except Croatia, which joined the EU in 2013. Out of 1895 Annex species that are required to be reported (see species code lists provided by the EEA^38^), we amassed occurrence records for 1695 reported species (including 41 marine species) in 10 km × 10 km grid cells across the EU, though 392 of these species did not occur in any PA that we considered here. Eventually, 1303 species were included in PA analyses: 469 birds, 105 fish, 93 mammals, 49 amphibians, 73 reptiles, 111 arthropods, 20 molluscs, one other invertebrate, 32 non-vascular and 350 vascular plants. We performed analyses across all taxa; see Supplementary Information for within-taxon analyses.

### Matching Species Distribution Data with Protected Areas

Since distribution data of reported species are variable in quality and have coarse spatial resolution, we applied a probabilistic approach for assigning each reported species to each PA, comparable to methods used in Araújo *et al*.^20^. When a reported species was present in several grid cells partially overlapping with the PA surface, the cumulative probability that the species was present in the PA was calculated by applying chain rule probability theory. The total probability of a species being present in a PA is, thus, the sum of all possible chain path probabilities that result in a probability of finding the species in the PA. In other words, the total probability of a species being present in a PA is the probability that a species is present in at least one of all overlapping parts between the PA and the occupied grid. Each chain step represents one particular grid cell containing a species that is partly covered by the PA. The total probability *p*_*i*_ of a species *i* being present in a single PA *j* is therefore calculated by

$${p}_{i;j}={c}_{1}+(1-{c}_{1}){c}_{2}+(1-{c}_{1})(1-{c}_{2}){c}_{3}+\cdots +(1-{c}_{1})(1-{c}_{2})\,\cdots \,(1-{c}_{n-1}){c}_{n}=1-(1-{c}_{1})(1-{c}_{2})(1-{c}_{3})\,\cdots \,(1-{c}_{n})=1-\prod _{k=1}^{n}(1-{c}_{k})$$

where *c* is the PA coverage of the *k*th of the *n* grid cells where the species is present. Imagine, for instance, two cells, 1 and 2, which record the presence of a given species. The PA covers 20% of cell 1 (c_1_ = 0.2) and 50% of cell 2 (c_2_ = 0.2). Therefore, the cumulative probability of finding the species within this PA is $1-(1-0.2)(1-0.5)=0.6$.

Each reported species was thus assigned to each PA with a probability of occurrence ranging from 0 to 1. We assume that at such an extensive scale, and for such large PAs, any bias or distortion of calculated species presence within PAs is acceptable, given the aims and scope of this study^53^. The limits of this approach are easily recognized, but it allows us to utilize one of the most fine-grained, freely available data sets that includes such a variety of taxa at a continental scale.

### Reported Species Richness and Adjustment for Area

We calculated the richness of reported species (*Richness_RS*) for each PA *j* as $Richness{\rm{\_}}R{S}_{j}={\sum }_{i=1}^{RS}\,{p}_{i;j}$ with *p*_*i;j*_ the probability to find the *i*^th^ species in the *j*^th^ PA. This represents the most likely number of reported species within each PA.

To account for the effect of PA size on *Richness_RS* we developed the *Richness_SAR_%Surplus* index, using the species–area relationship (SAR). *Richness_SAR_%Surplus* measures the number of species present in excess of the richness expected from the best-fitting SAR model, expressed as a percentage of the expected richness. SAR modelling has rarely been used for evaluating protected areas (but see Chiarucci *et al*.^34^).

We modelled species–area relationships (SARs) by fitting the classic Arrhenius Power Function^54^, $Richness{\rm{\_}}RS=b\cdot {A}^{c}$, and Gleason’s Exponential Model^55^, $Richness{\rm{\_}}RS=y+z\cdot log(A)$. In these models, *b* and *y* represent the number of species expected for unit area, while *c* and *z* represent the increase in the number of species with surface area (with different scaling of area in the two models). We fitted the models for the whole set of PAs for which data were available and compared them to a linear null model with intercept equals 0. We performed SAR model selection using Akaike’s Information Criterion (AIC), suggested to be one of the most appropriate statistical methods for comparing such models^56^. The Arrhenius model fitted best (i.e. lowest AIC; Supplementary Fig. S13) and was used as the SAR reference model for calculating the expected value of *Richness_RS*; the residuals of the SAR model were divided by the corresponding fitted values and multiplied by 100. The resulting value is therefore the number of species in the PA over and above the number expected from the species–area relationship, expressed as a percentage of the expected number; it can be labelled the ‘area-controlled surplus of reported species’ (abbreviated as *Richness_SAR_%Surplus*). Positive values indicate PAs with more species than expected under the SAR model, and negative values indicate fewer than expected.

### Conservation Weight of Reported Species

The measures described above (*Richness_RS* and *Richness_SAR_%Surplus*) assign equal importance to all reported species. To provide a better quantification of the conservation value of each PA, we calculated a conservation weight *w*_*i*_, for each species *i*, as the inverse of the sum of grid cells occupied by the species in the land area of the EU^57^. In this study, the conservation weight is also referred to as ‘rarity’. The more grid cells the species is present in, the lower is its rarity and, respectively, its conservation weight.

### Rarity-weighted Richness and Average Rarity

Rarity-weighted richness of each PA *j* was calculated by $\,Richness\_Rarity\_weighte{d}_{j}={\sum }_{i=1}^{RS}{p}_{i;j}\cdot {w}_{i}$ where *p*_*i;j*_ represents the likely presence of each reported species in the *j*^th^ PA and *w*_*i*_ is the conservation weight of the species. This index becomes 0 when no reported species are present in the PA. It increases the more species the PA contains and the higher their conservation weights are. Thus, *Richness_Rarity_weighted* considers species richness and rarity simultaneously.

Since *Richness_Rarity_weighted* is partly dependent on the number of reported species within the PA, we introduce another index, the average rarity (*Rarity_Mean*). This measures the average conservation weight of reported species within the PA, and is calculated as *Richness_Rarity_weighted*/*Richness_RS*. It is set to 0 when the PA contains no reported species, and has maximum value 1. It reaches its maximum value of 1 when all the reported species in the PA are present with probability 1 and each only occurs in one grid cell that is entirely within the PA. When *Rarity_Mean* is 1, the PA is absolutely unique for preserving the set of species.

### Differentiation Diversity

Differentiation diversity (beta diversity) between PAs gives additional information about the conservation value of PAs^43,44^. If a PA’s species composition is very similar to others, it is less unique. We used Baselga’s concept of beta diversity^58^ ─ adapting Bray-Curtis dissimilarity index and its components ─ to measure the dissimilarity between sets of species occurrence probabilities of PAs. The beta diversity metrics return a dissimilarity value between two numerical vectors of the same length, no matter the meaning of the number (abundance or probability). This enables the use of the occurrence probabilities as input values for these indices, rather than forcing the probabilities into values from 1 to 0 to estimate abundances, which would implicate additional uncertainty. Thus, a dissimilarity value of 0 means the same composition of the species’ occurrence probabilities, and a value of 1 indicates totally different composition of probabilities. In Baselga’s concept^58^ the total dissimilarity (*Dissimilarity_Total*) is additively partitioned into the balanced (*Dissimilarity_Balanced*, i.e. turnover) and gradient (*Dissimilarity_Gradient*, i.e. nestedness) components, allowing a more sophisticated assessment of the PAs’ conservation value. Balanced dissimilarity, *Dissimilarity_Balanced*, is equivalent to turnover between two sample sites and quantifies, in our case, a balanced change of occurrence probabilities between sites: some species gain in probability of occurrence, while others lose. Accordingly, gradient dissimilarity, *Dissimilarity_Gradient*, is equivalent to nestedness and represents monotonic increase or decrease (i.e. a gradient) of occurrence probabilities between sample sites. In order to calculate the compositional uniqueness of a given PA to all other PAs, we took the mean of all pairwise dissimilarities of the PA compared with all others. To detect the variation of pairwise dissimilarities per PA, we also calculated the standard deviation (Supplementary Fig. S2).

### Sensitivity analyses

We applied sensitivity analyses to estimate the potential bias of species reporting by individual EU member states and its effect on uniqueness values of PAs. The EU nations are obliged to report on each Annex species individually. We assume that a potential bias was induced by each Annex species present in, but not reported by, EU states. We do not assume that the nations’ reported species distributions are biased, so the species’ conservation weights are the best estimate of the species’ real rarity in the EU. Therefore we maintained the weights in the sensitivity analyses. The sensitivity analyses comprise three approaches. First, we followed a null model approach to test whether observed conservation values of PAs (based on the original data) are significantly (i.e. *p* < 0.05) different from values randomly expected (based on simulated data). Regarding the simulated data, we randomly distributed the species a thousand times (1,000 random simulations), maintaining the frequencies of each species, i.e. the species’ conservation weights in the EU. We thus simulate a quite arbitrary reporting of species occurrences in grid cells and respectively PAs that is only restricted to the species that were originally reported, and to their total frequencies. We recalculated the metrics of conservation value for each PA and simulation run. We tested for significant differences between the observed values and the randomized values within each nation via the non-parametric, two-sided and paired Wilcoxon signed rank test. Second, we used the non-parametric, two-sided but unpaired Wilcoxon rank sum test (Mann-Whitney test) to check for significant differences between the observed values within each nation and the observed values within rest of the EU. Thus we can estimate whether the observed conservation values of PAs within nations are rather usual or extreme (low or high) with respect to, first, arbitrary reporting and, second, conservation values at an EU-average (of all other EU nations). Third, we conducted a cross-validation procedure that accounts for the uncertainty of resulting conservation values under potential reporting bias. Here we also ran 1,000 simulations. In each simulation run, we randomly chose 20 out of 25 nations without replacement. We thus simulate reporting deficits by excluding reported species and PAs of five nations. We decided to exclude five countries, since the official data quality evaluation of the EEA^45^ highlights five nations of serious reporting deficiency. Based on the remaining PAs we recalculated *Richness_SAR_%Surplus* and the *Dissimilarity* indices. Other indices are not affected by this simulated lack of species reporting, since the conservation weights are kept constant. We took the standard deviation (SD) of the 1,000 runs as a measure of uncertainty and, respectively, non-robustness of conservation values against bias.

### Irreplaceability and the Metrics of Conservation Value

Our metrics of conservation value are summarized in Table 1. We investigated correlations among the metrics, and between each of these and the PAs’ irreplaceability scores as calculated by Le Saout *et al*.^23^ (Fig. 3). We used Pearson correlation and a modified t-test that accounts for spatial autocorrelation^59^, to derive the *p*-values. Le Saout *et al*.^23^ measured irreplaceability by the overlap of each PA with species’ ranges (rather than occurrence records, as here) of a subset of the taxa that we include (amphibian, mammal and bird species from the IUCN Red List of Threatened Species). We used ArcGIS (Version 10.3.1; ESRI, Redlands, CA) and R^60^ (Version 3.3.3) for the geospatial and statistical analyses.

### Data availability

Datasets analyzed in the current study are publicly available. Data on PAs are obtained from the World Database on Protected Areas^51^ and the UNESCO MAB Biosphere Reserve Directory^40^. We used species’ occurrence data published by the European Environment Agency in fulfilment of EU legislation^38^. Irreplaceability scores of PAs are taken from Le Saout *et al*.^23^.

## Electronic supplementary material

Supplementary Information

Dataset 1
