# Supplementary material for: Uniqueness of Protected Areas for Conservation Strategies in the European Union
Source: Sci Rep. 2018 Apr 24;8:6445. doi: 10.1038/s41598-018-24390-3 (PMC5915414; doi:10.1038/s41598-018-24390-3)
Supplement: Supplementary file 1 — Supplementary Information [file 41598_2018_24390_MOESM1_ESM.docx]

**Supplementary Information to**

***Uniqueness of Protected Areas for Conservation Strategies in the European Union***

**Authors**

Samuel Hoffmann^1*^, Carl Beierkuhnlein^1^, Richard Field^2^, Antonello Provenzale^3^, Alessandro Chiarucci^4^

**Affiliations and Addresses**

^1^ Department of Biogeography, BayCEER, University of Bayreuth, D-95440, Bayreuth, Germany; [samuel.hoffmann@uni-bayreuth.de](mailto:samuel.hoffmann@uni-bayreuth.de), ORCID ID: 0000-0002-6176-8406; [carl.beierkuhnlein@uni-bayreuth.de](mailto:carl.beierkuhnlein@uni-bayreuth.de), ORCID ID: 0000-0002-6456-4628;

^2^ School of Geography, University of Nottingham, University Park, NG7 2RD, UK; richard.field@nottingham.ac.uk; ORCID ID: 0000-0003-2613-2688

^3^ Institute of Geosciences and Earth Resources, National Research Council of Italy, Via Moruzzi 1, 56124 Pisa, Italy; email: [antonello.provenzale@cnr.it](mailto:antonello.provenzale@cnr.it); ORCID ID: [0000-0003-0882-5261](http://orcid.org/0000-0003-0882-5261)

^4^ Department of Biological, Geological, and Environmental Sciences, Alma Mater Studiorum – University of Bologna, Via Irnerio 42, 40126 Bologna, Italy; [alessandro.chiarucci@unibo.it](mailto:alessandro.chiarucci@unibo.it); ORCID ID: 0000-0003-1160-235X

* Corresponding author: Department of Biogeography, BayCEER, University of Bayreuth, Universitaetsstr. 30, D-95440, Bayreuth, Germany; Tel.: +49 921 552211, Fax: +49 921 552315, samuel.hoffmann@uni-bayreuth.de

*Supplementary Figures*


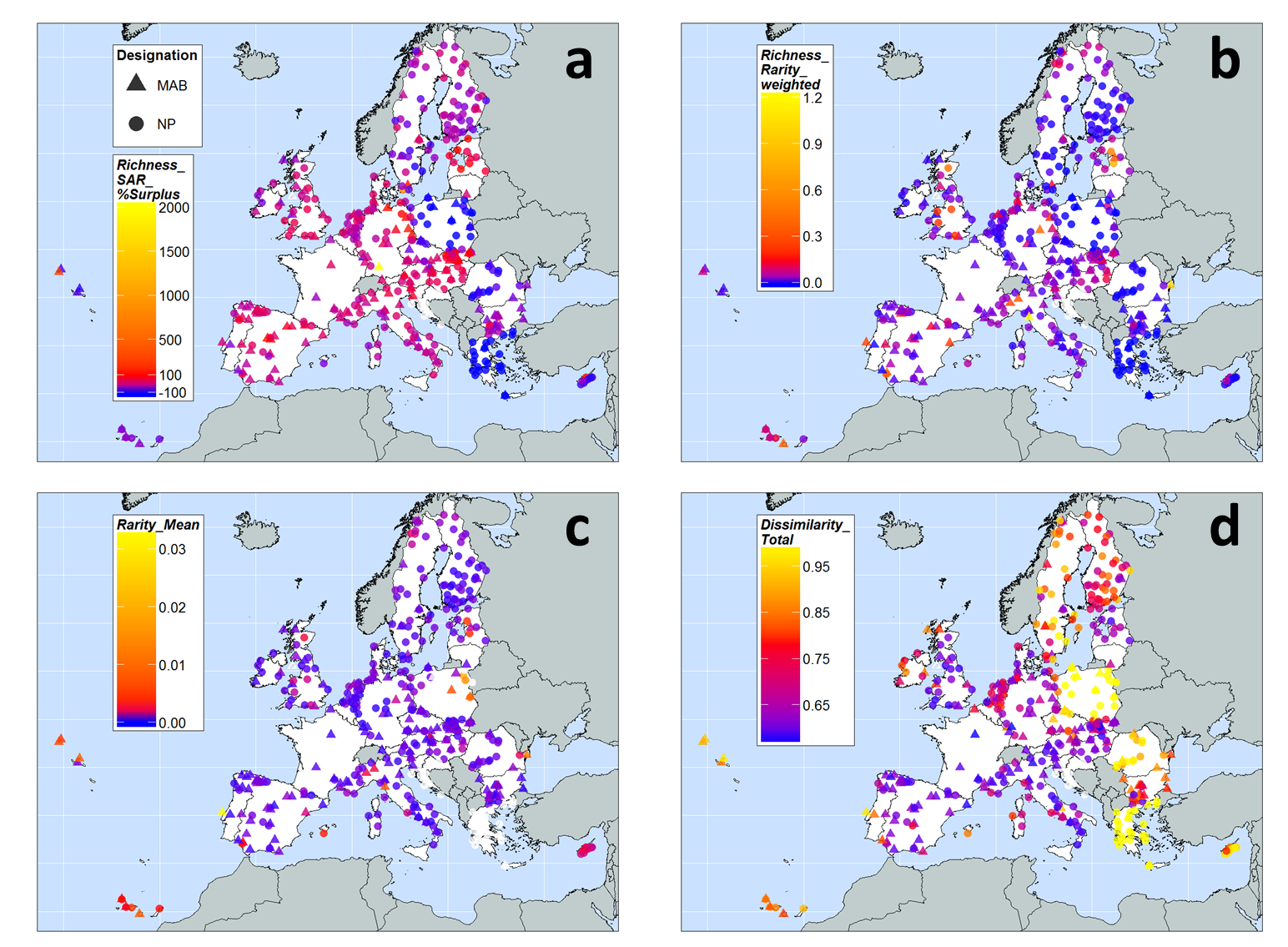


Figure S1: Uniqueness indices of protected areas calculated separately for 469 birds of the Birds Directive. a) Area-controlled surplus of reported species *Richness_SAR_%Surplus*. b) Rarity-weighted richness *Richness_Rarity_weighted.* c) Average rarity *Rarity_Mean.* d) Total dissimilarity *Dissimilarity_Total*. For details about indices’ definition see Methods section. White symbols illustrate missing data. MAB: Man and Biosphere Reserve; NP = National Park. The maps were created using open-source software R, Version 3.3.3 (<https://www.R-project.org/>) ^[60]^.


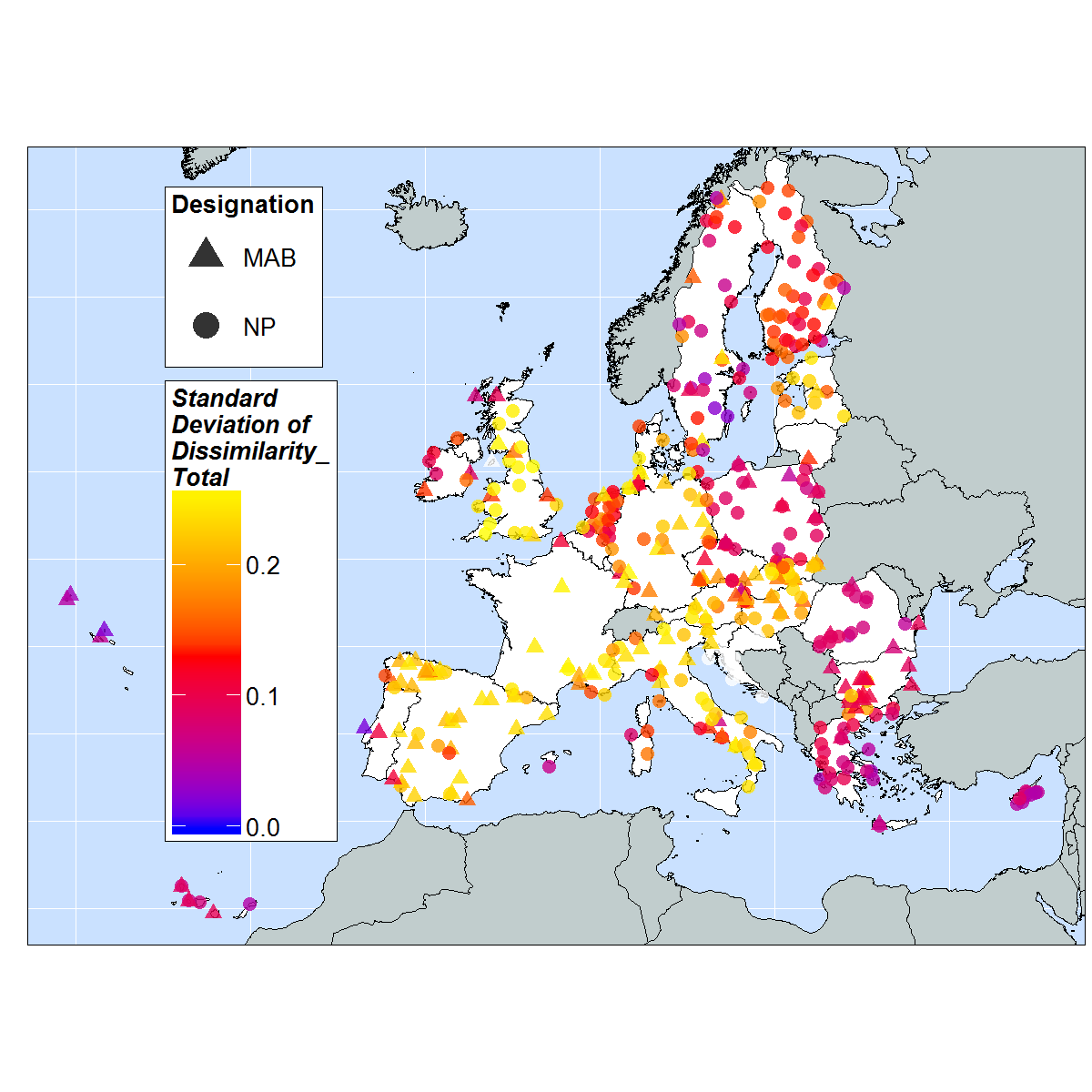


Figure S2: The standard deviation of the pairwise total dissimilarity values calculated for each national park (NP) and Man and Biosphere Reserve (MAB). The mean of these pairwise total dissimilarity values results in the total dissimilarity index *(Dissimilarity_Total)* per protected area. For details about indices’ definition see Methods section in main text. White symbols illustrate missing data. The map was created using open-source software R, Version 3.3.3 (<https://www.R-project.org/>) ^[60]^.


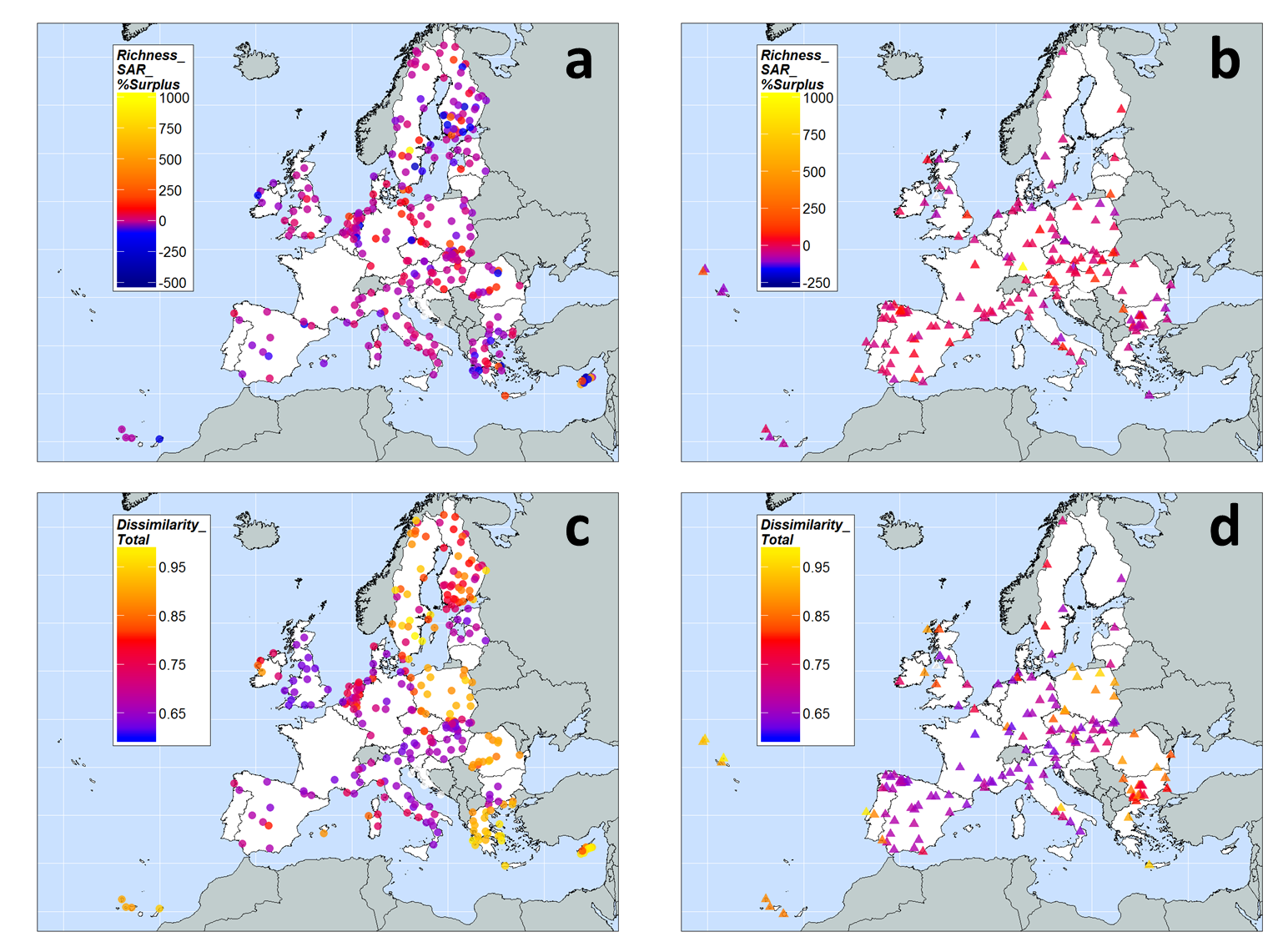


Figure S3: Area-controlled surplus of reported species (*Richness_SAR_%Surplus*) and total dissimilarity *(Dissimilarity_Total)* calculated separately for the national park (NP) and Man and Biosphere Reserve (MAB) network. a) *Richness_SAR_%Surplus* values calculated for the NP network differ from NP values of the combined network (paired Wilcoxon test: *p*=0.03), but the geographic pattern is quite similar (Pearson correlation coefficient *r*=0.23). b) The same is true for *Richness_SAR_%Surplus* of the MAB network (*p*<0.01, *r*=0.88). c) *Dissimilarity_Total* of the NP network (*p*<0.01, *r*=1.00). d) *Dissimilarity_Total* of the MAB network (*p*<0.01, *r*=0.99). For details about indices’ definition see Methods section in main text. White symbols illustrate missing data. The maps were created using open-source software R, Version 3.3.3 (<https://www.R-project.org/>) ^[60]^.


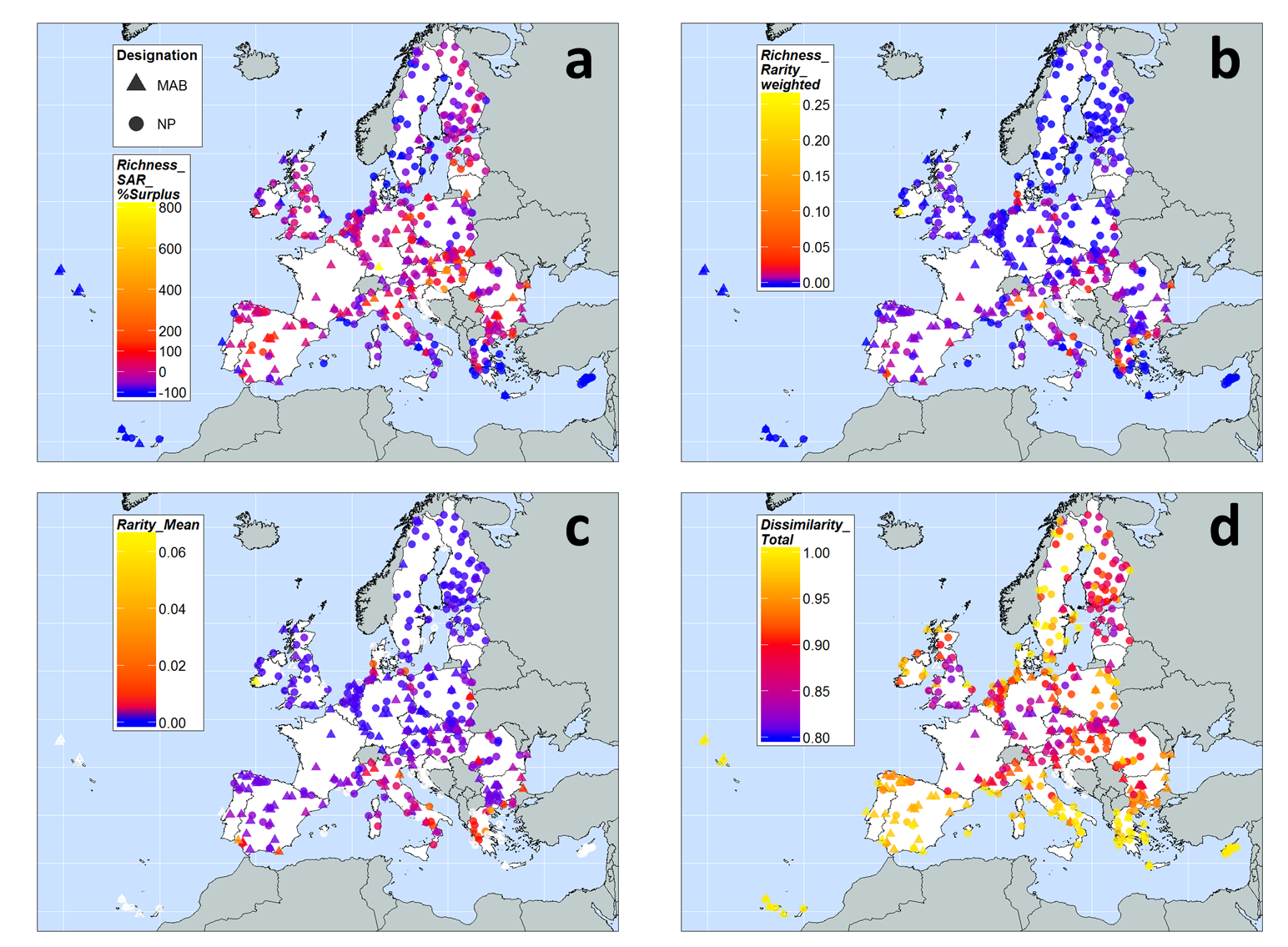


Figure S4: Uniqueness indices of protected areas calculated separately for 105 fish of the Habitats Directive. a) Area-controlled surplus of reported species *Richness_SAR_%Surplus*. b) Rarity-weighted richness *Richness_Rarity_weighted.* c) Average rarity *Rarity_Mean.* d) Total dissimilarity *Dissimilarity_Total*. For details about indices’ definition see Methods section. White symbols illustrate missing data. MAB: Man and Biosphere Reserve; NP = National Park. The maps were created using open-source software R, Version 3.3.3 (<https://www.R-project.org/>) ^[60]^.


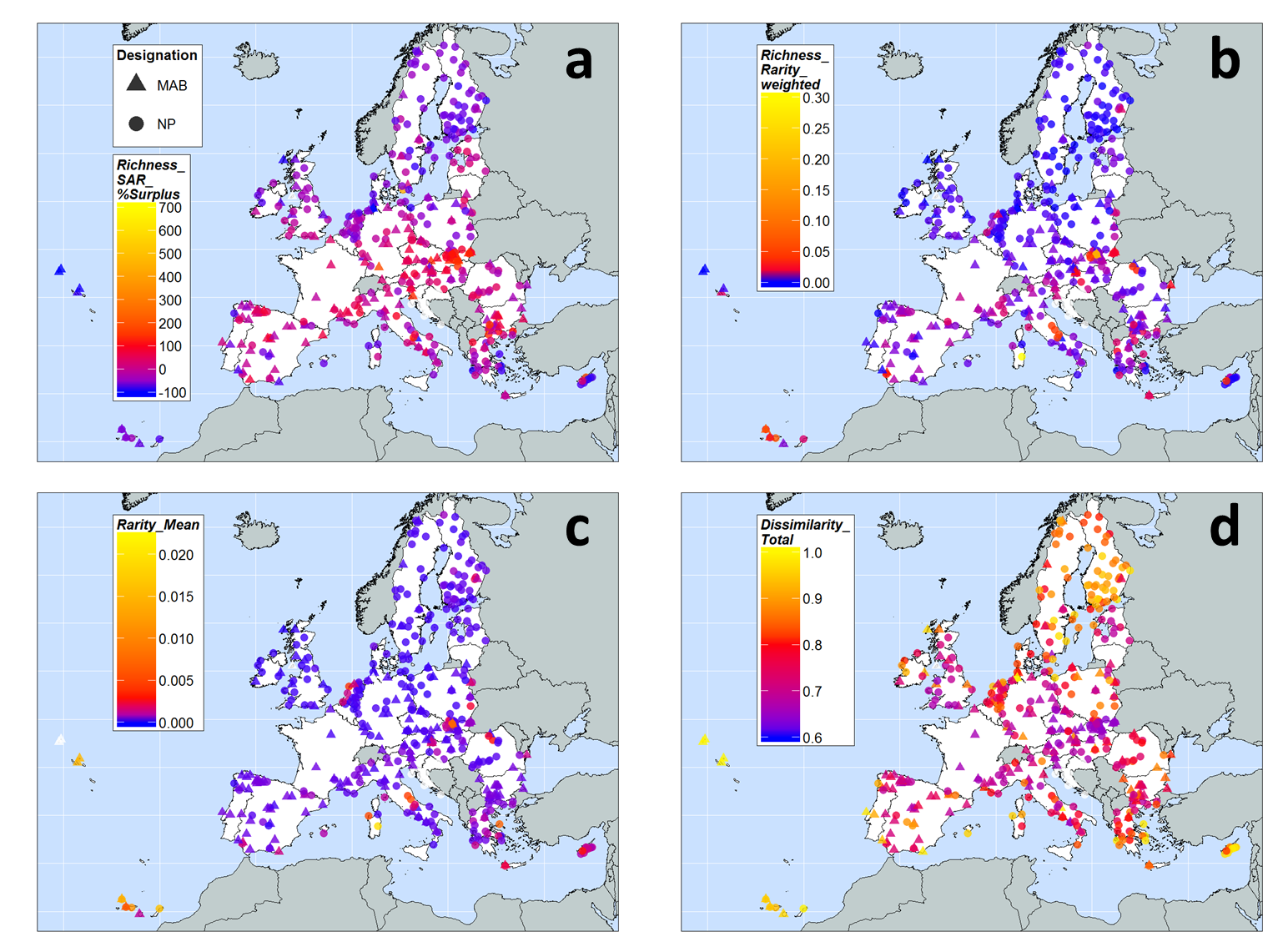


Figure S5: Uniqueness indices of protected areas calculated separately for 93 mammals of the Habitats Directive. a) Area-controlled surplus of reported species *Richness_SAR_%Surplus*. b) Rarity-weighted richness *Richness_Rarity_weighted.* c) Average rarity *Rarity_Mean.* d) Total dissimilarity *Dissimilarity_Total*. For details about indices’ definition see Methods section. White symbols illustrate missing data. MAB: Man and Biosphere Reserve; NP = National Park. The maps were created using open-source software R, Version 3.3.3 (<https://www.R-project.org/>) ^[60]^.


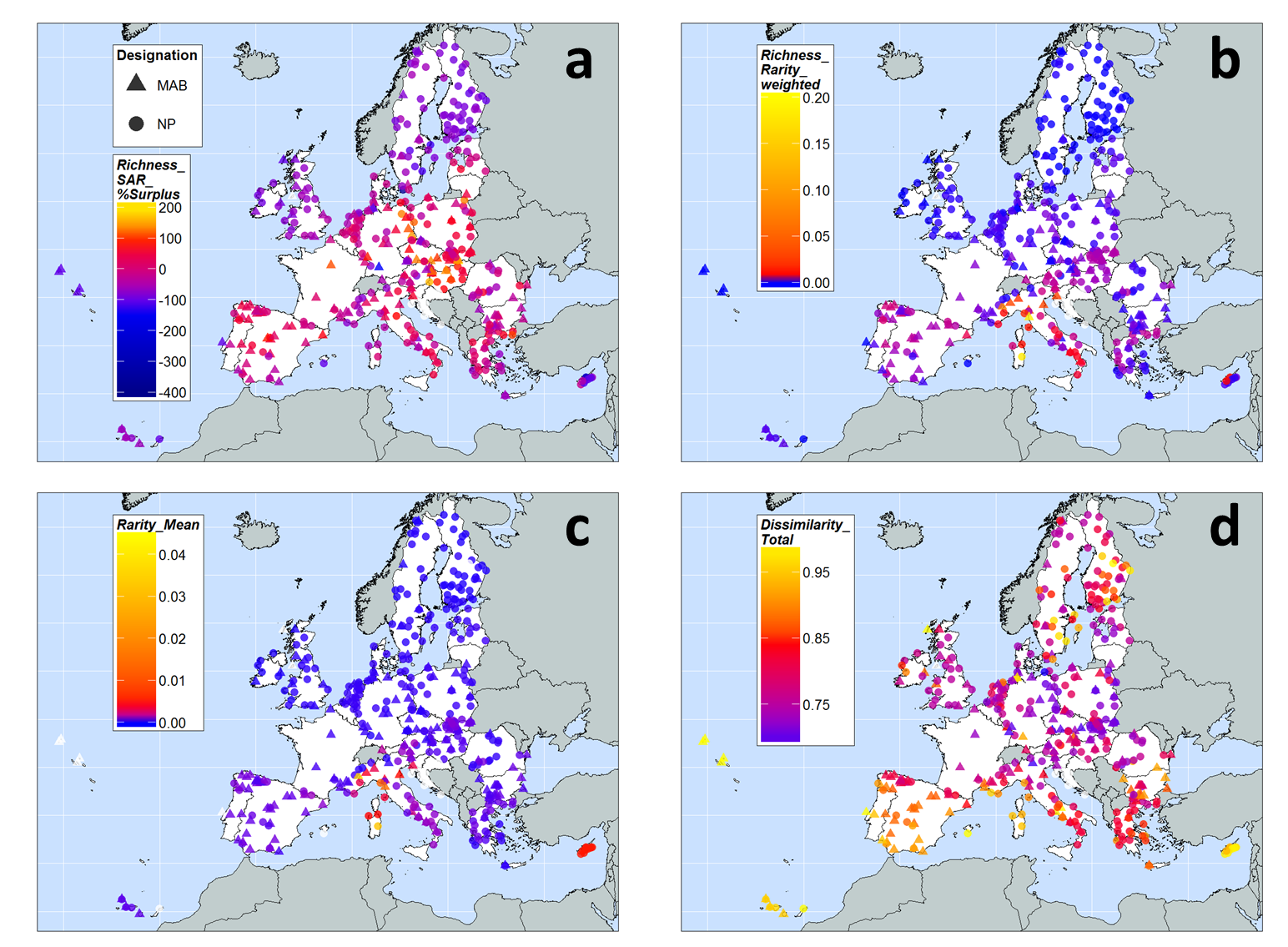


Figure S6: Uniqueness indices of protected areas calculated separately for 49 amphibians of the Habitats Directive. a) Area-controlled surplus of reported species *Richness_SAR_%Surplus*. b) Rarity-weighted richness *Richness_Rarity_weighted.* c) Average rarity *Rarity_Mean.* d) Total dissimilarity *Dissimilarity_Total*. For details about indices’ definition see Methods section. White symbols illustrate missing data. MAB: Man and Biosphere Reserve; NP = National Park. The maps were created using open-source software R, Version 3.3.3 (<https://www.R-project.org/>) ^[60]^.


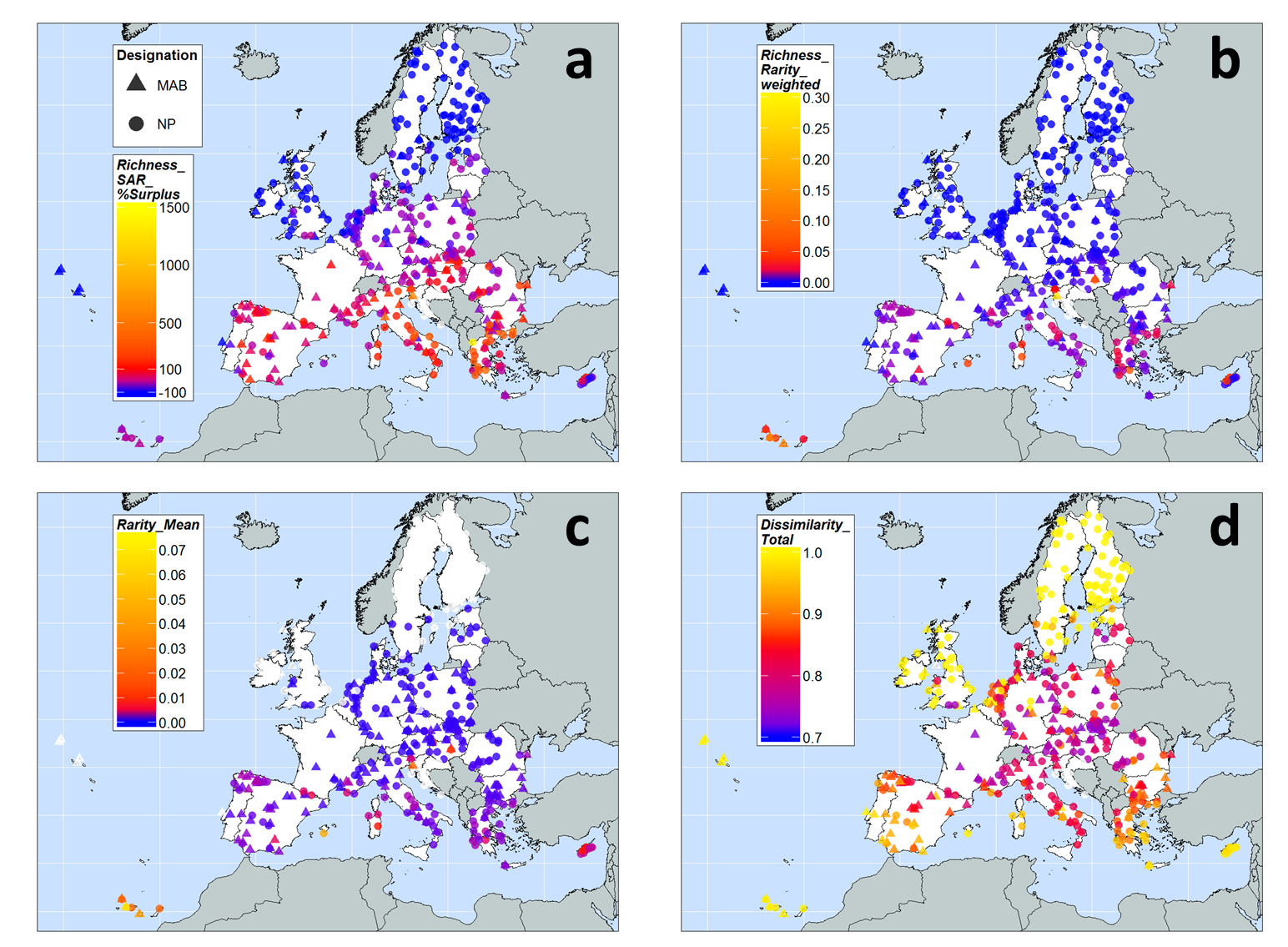
Figure S7: Uniqueness indices of protected areas calculated separately for 73 reptiles of the Habitats Directive. a) Area-controlled surplus of reported species *Richness_SAR_%Surplus*. b) Rarity-weighted richness *Richness_Rarity_weighted.* c) Average rarity *Rarity_Mean.* d) Total dissimilarity *Dissimilarity_Total*. For details about indices’ definition see Methods section. White symbols illustrate missing data. MAB: Man and Biosphere Reserve; NP = National Park. The maps were created using open-source software R, Version 3.3.3 (<https://www.R-project.org/>) ^[60]^.


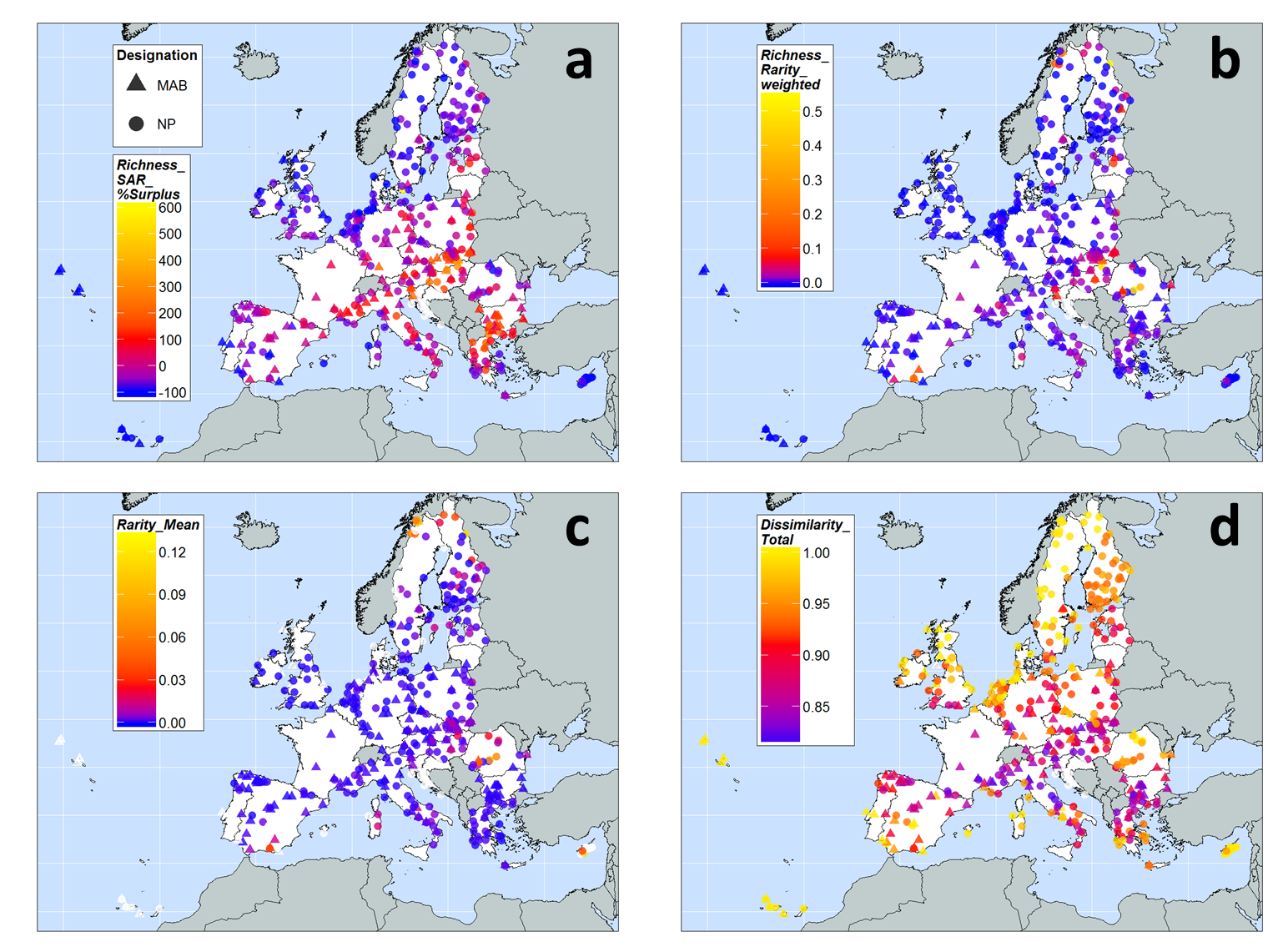


Figure S8: Uniqueness indices of protected areas calculated separately for 111 arthropods of the Habitats Directive. a) Area-controlled surplus of reported species *Richness_SAR_%Surplus*. b) Rarity-weighted richness *Richness_Rarity_weighted.* c) Average rarity *Rarity_Mean.* d) Total dissimilarity *Dissimilarity_Total*. For details about indices’ definition see Methods section. White symbols illustrate missing data. MAB: Man and Biosphere Reserve; NP = National Park. The maps were created using open-source software R, Version 3.3.3 (<https://www.R-project.org/>) ^[60]^.


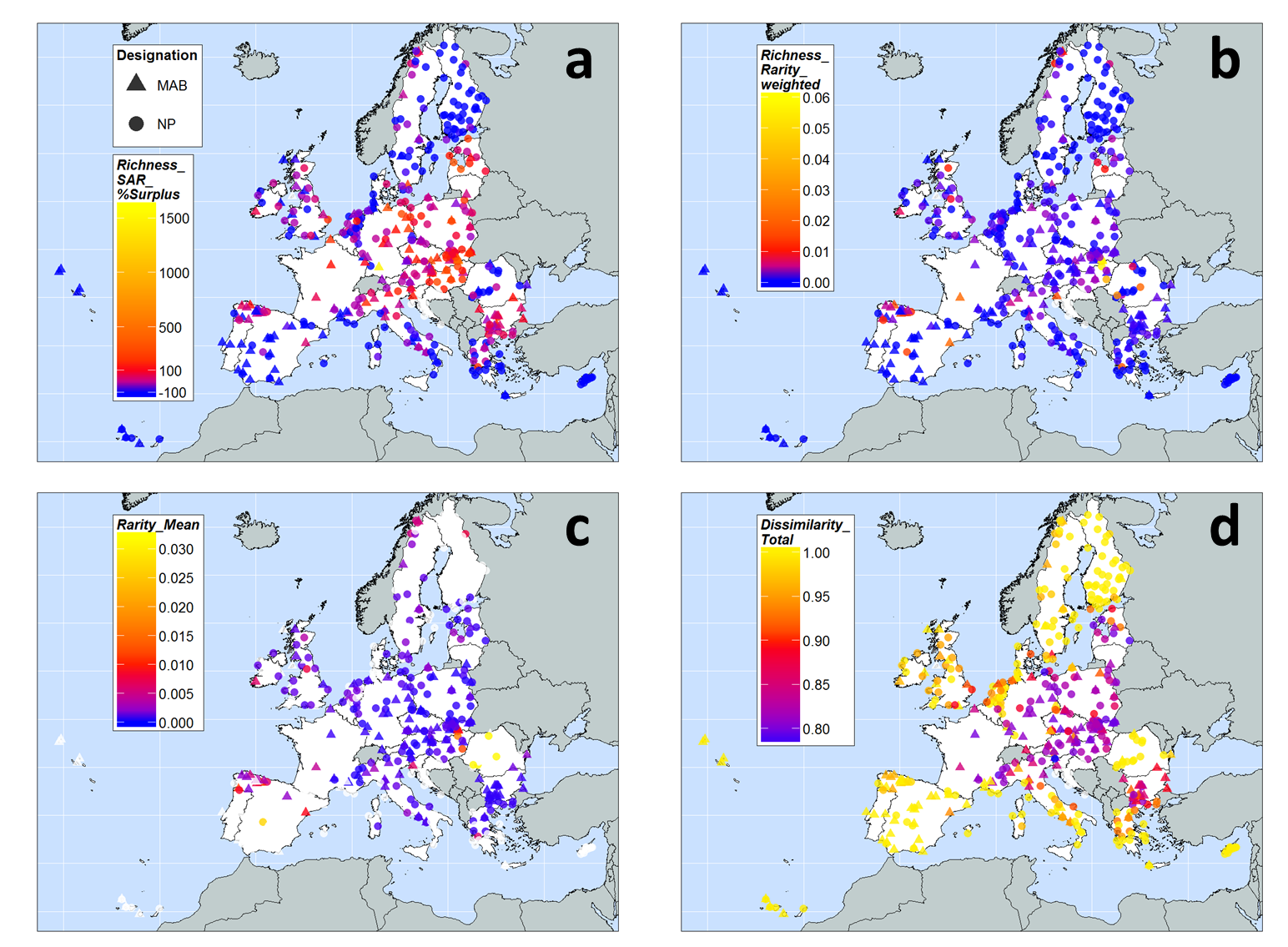


Figure S9: Uniqueness indices of protected areas calculated separately for 20 molluscs of the Habitats Directive. a) Area-controlled surplus of reported species *Richness_SAR_%Surplus*. b) Rarity-weighted richness *Richness_Rarity_weighted.* c) Average rarity *Rarity_Mean.* d) Total dissimilarity *Dissimilarity_Total*. For details about indices’ definition see Methods section. White symbols illustrate missing data. MAB: Man and Biosphere Reserve; NP = National Park. The maps were created using open-source software R, Version 3.3.3 (<https://www.R-project.org/>) ^[60]^.


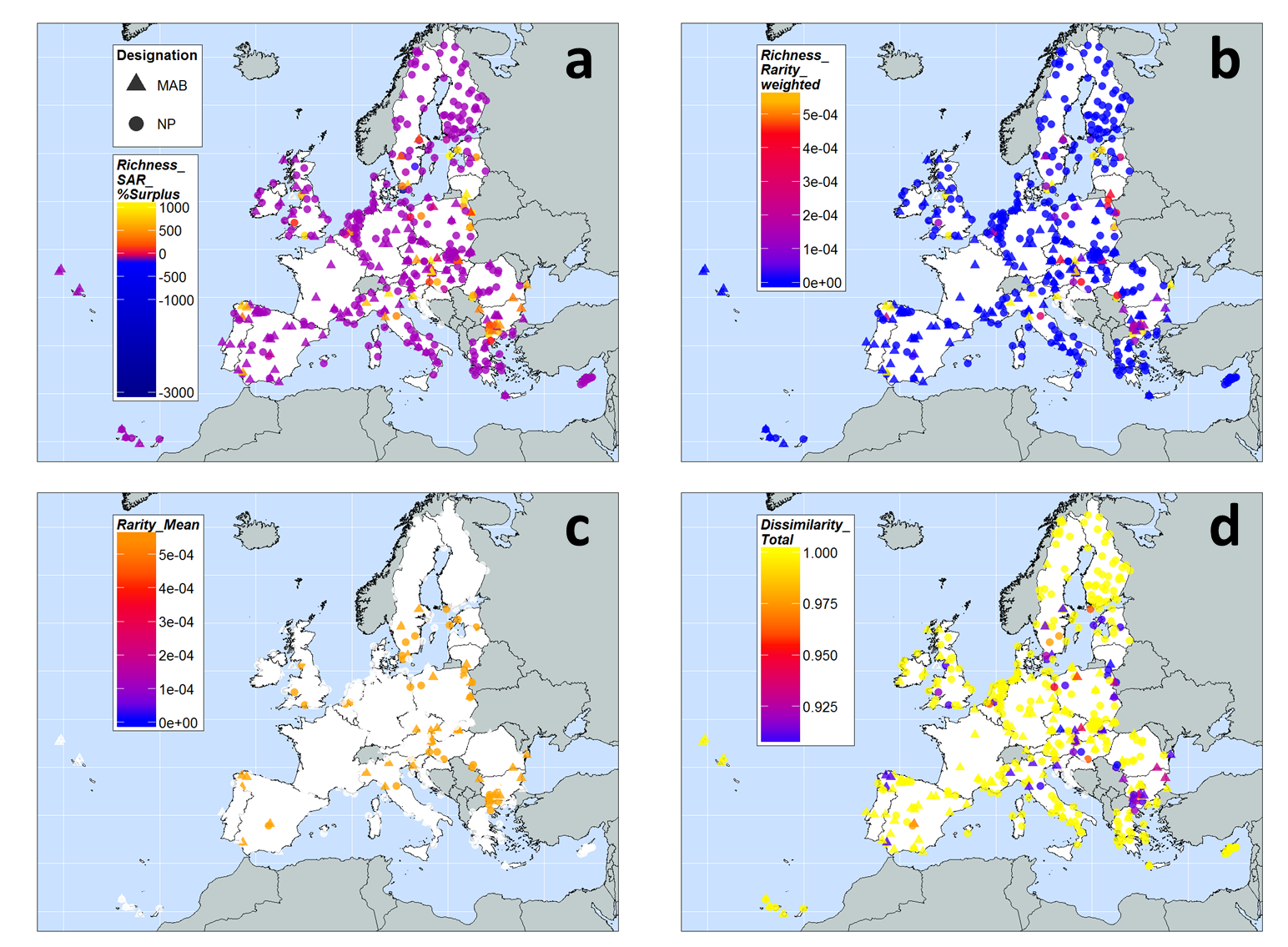


Figure S10: Uniqueness indices of protected areas calculated separately for one other invertebrate of the Habitats Directive. a) Area-controlled surplus of reported species *Richness_SAR_%Surplus*. b) Rarity-weighted richness *Richness_Rarity_weighted.* c) Average rarity *Rarity_Mean.* d) Total dissimilarity *Dissimilarity_Total*. For details about indices’ definition see Methods section. White symbols illustrate missing data. MAB: Man and Biosphere Reserve; NP = National Park. The maps were created using open-source software R, Version 3.3.3 (<https://www.R-project.org/>) ^[60]^.


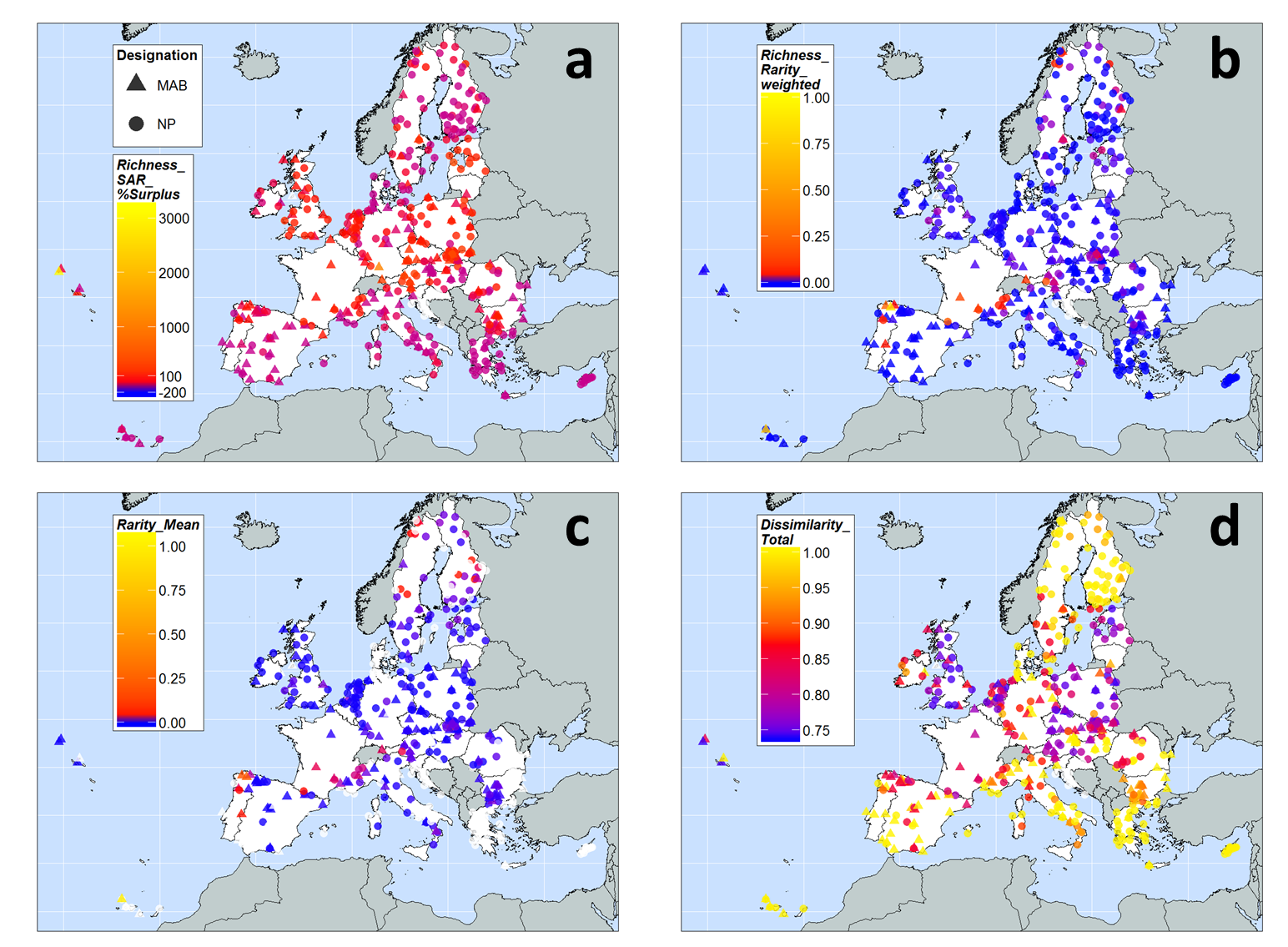


Figure S11: Uniqueness indices of protected areas calculated separately for 32 non-vascular plants of the Habitats Directive. a) Area-controlled surplus of reported species *Richness_SAR_%Surplus*. b) Rarity-weighted richness *Richness_Rarity_weighted.* c) Average rarity *Rarity_Mean.* d) Total dissimilarity *Dissimilarity_Total*. For details about indices’ definition see Methods section. White symbols illustrate missing data. MAB: Man and Biosphere Reserve; NP = National Park. The maps were created using open-source software R, Version 3.3.3 (<https://www.R-project.org/>) ^[60]^.


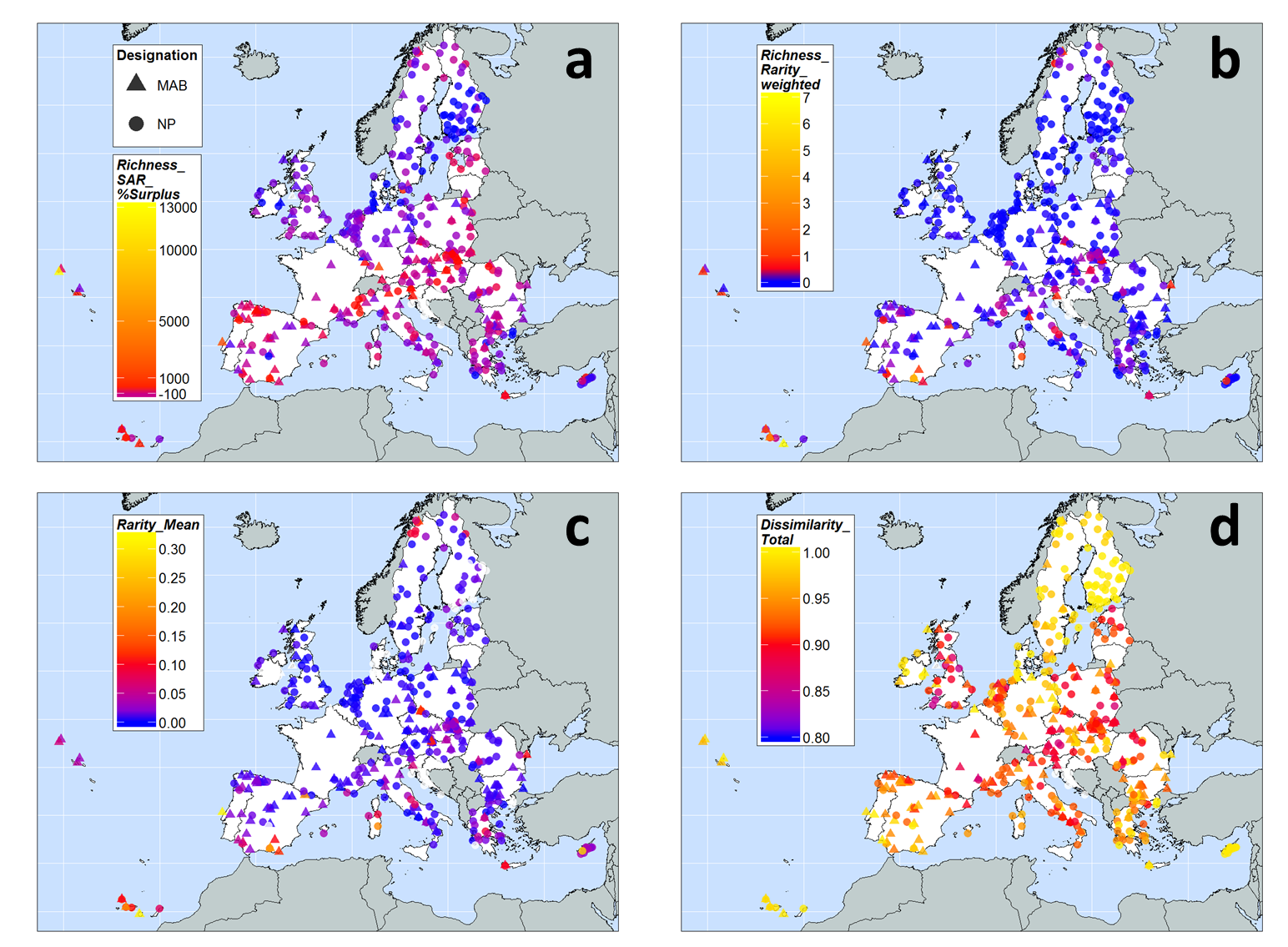


Figure S12: Uniqueness indices of protected areas calculated separately for 350 vascular plants of the Habitats Directive. a) Area-controlled surplus of reported species *Richness_SAR_%Surplus*. b) Rarity-weighted richness *Richness_Rarity_weighted.* c) Average rarity *Rarity_Mean.* d) Total dissimilarity *Dissimilarity_Total*. For details about indices’ definition see Methods section. White symbols illustrate missing data. MAB: Man and Biosphere Reserve; NP = National Park. The maps were created using open-source software R, Version 3.3.3 (<https://www.R-project.org/>) ^[60]^.


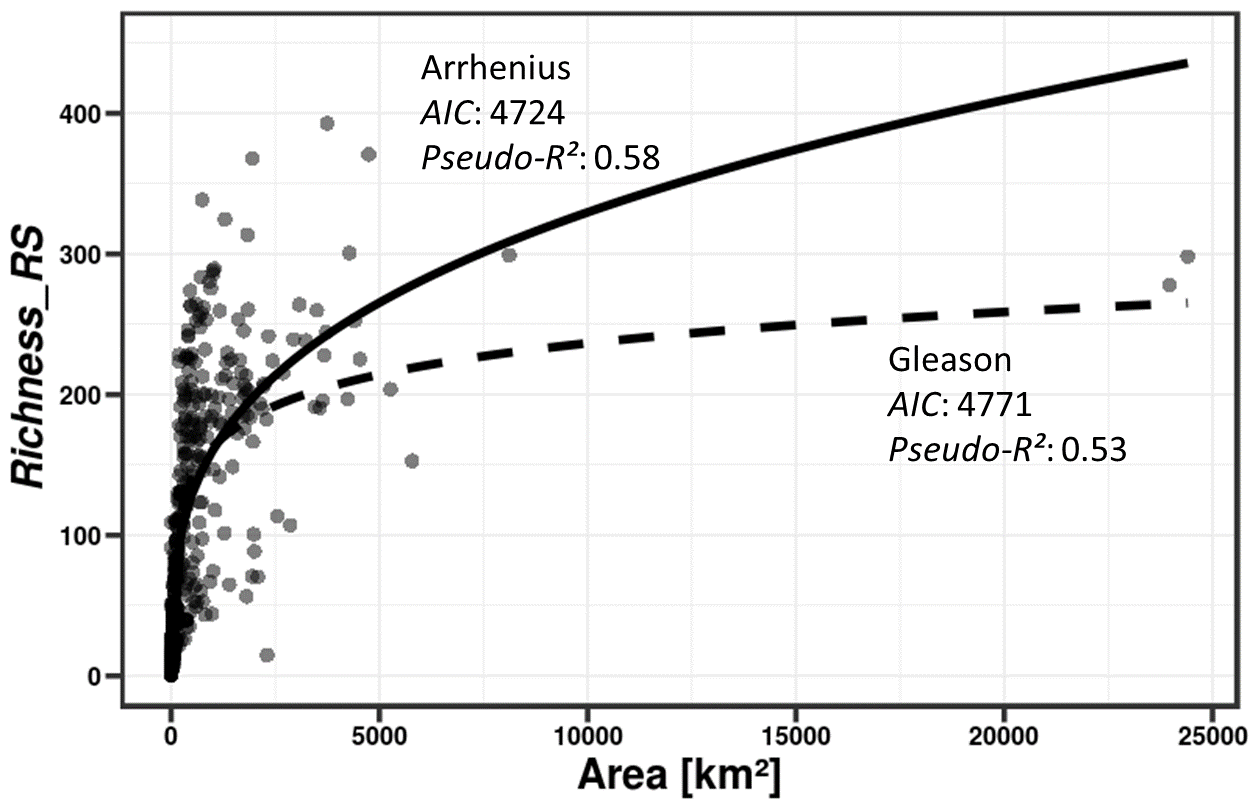


Figure S13: The species-area relationships (SAR) adpated to the set of protected areas (national parks and biosphere reserves) and reported species richness (*Richness_RS*). We compared the Arrhenius and Gleason model to a null model, i.e. the intercept-only model. The *Pseudo-R²* is calculated by 1-(*Model Deviance*/*Null Model Deviance*). The Arrhenius model is fitting best (i.e. lowest *AIC*) and was therefore chosen to calculate *Richness_SAR_%Surplus*. For details see methods section.
